# Targeting Macrophage STING *O*‐GlcNAcylation via Small‐Molecule Allosteric Modulation for Psoriasis Therapy

**DOI:** 10.1002/advs.78257

**Published:** 2026-10-14

**Authors:** Fang‐Fang Zhuo, Cheng‐Chen Hou, Wei Wang, Ya‐Xuan Zhu, Ling Li, Li‐Juan Liu, Chuan‐Jian Lu, Peng‐Fei Tu, Yang Liu, Rui‐Zhi Zhao, Ke‐Wu Zeng

**Affiliations:** ^1^ State Key Laboratory of Natural and Biomimetic Drugs School of Pharmaceutical Sciences Peking University Beijing China; ^2^ State Key Laboratory of Dampness Syndrome of Chinese Medicine The Second Affiliated Hospital of Guangzhou University of Chinese Medicine Guangzhou China; ^3^ Center of Basic Medical Research Institute of Medical Innovation and Research Peking University Third Hospital Beijing China; ^4^ Department of Integration of Chinese and Western Medicine School of Basic Medical Sciences Peking University Beijing China; ^5^ Department of Integrated Traditional Chinese and Western Medicine Peking University First Hospital Beijing China

**Keywords:** allosteric rearrangement, cyclic dinucleotide‐binding domain, paeoniflorin, psoriasis, STING *O*‐GlcNAcylation

## Abstract

STING undergoes distinct post‐translational modifications in autoimmune disorders, particularly in psoriasis. However, selectively targeting these disease‐associated STING modifications remains a major therapeutic challenge. Here, we show that macrophage STING *O*‐GlcNAcylation is a critical driver of psoriatic inflammation and identify the natural small‐molecule paeoniflorin (PF) as a selective inhibitor of STING *O*‐GlcNAcylation. Mechanistically, PF engages the cyclic dinucleotide‐binding domain (CBD) of STING, inducing an allosteric conformational shift that traps STING in an inactive open state. This allosteric mechanism sterically blocks *O*‐GlcNAc transferase (OGT) recruitment to STING, thereby suppressing STING *O*‐GlcNAcylation and subsequent TBK1‐dependent phosphorylation cascades. In vivo, PF markedly alleviates psoriatic skin inflammation, whereas myeloid‐specific STING ablation substantially compromises its therapeutic efficacy. Critically, clinical analyses of patient‐derived samples further support the relevance of STING *O*‐GlcNAcylation to psoriasis. Together, our work uncovers macrophage STING *O*‐GlcNAcylation as a critical regulatory checkpoint in psoriatic inflammation, highlighting a promising natural product‐based strategy for targeting pathological STING signaling.

## Graphical abstract

Paeoniflorin allosterically targets the STING cyclic dinucleotide‐binding domain, stabilizes an inactive open conformation, and disrupts STING–OGT interaction to reduce Thr229 *O*‐GlcNAcylation and downstream signaling. This mechanism restrains myeloid STING activation, alleviates psoriasis‐like inflammation, and is supported by analyses of patient‐derived samples, highlighting the clinical relevance of STING *O*‐GlcNAcylation in psoriasis.

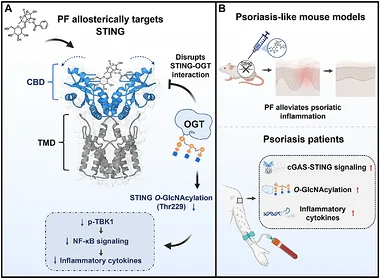

## 1 Introduction

Psoriasis, a chronic autoimmune dermatologic disorder, is characterized by inflammatory lesions with infiltration of diverse immune cell populations, resulting in epidermal hyperplasia, dysregulated keratinocyte proliferation, and systemic inflammation [1, 2]. The stimulator of interferon genes (STING) pathway is a central hub in innate immune signaling and has emerged as a key driver of psoriatic inflammation [3, 4]. However, the development of STING‐targeted therapeutics remains a major challenge because of the suboptimal selectivity of currently available small‐molecule compounds [5, 6]. STING activity is tightly regulated by multiple post‐translational modifications (PTMs) [7, 8], highlighting the complexity of its activation and signaling control. Accordingly, current STING‐targeted strategies remain limited by insufficient selectivity, raising concerns about off‐target toxicity and unintended immunosuppressive effects [9]. Therefore, identifying small molecules that selectively modulate specific PTM‐associated regulatory events of STING may offer a more precise therapeutic strategy for autoimmune disorders.

STING activity is precisely regulated through multiple PTMs, including phosphorylation [10], ubiquitination [11], palmitoylation [12], and *O*‐GlcNAcylation [13]. These PTMs orchestrate a dynamic regulation of STING signaling and contribute to pathological immune dysregulation, which is characterized by sustained inflammation in autoimmune pathogenesis [14]. In particular, *O*‐GlcNAcylation has recently emerged as a research hotspot due to its critical roles in regulating STING stability, subcellular localization, and signal transduction [15, 16]. However, current research on small‐molecule modulators of STING *O*‐GlcNAcylation remains preliminary, with limited mechanistic understanding of how these compounds regulate *O*‐GlcNAcylation at the molecular level. This limited mechanistic insight not only constrains our understanding of biological roles in STING *O*‐GlcNAcylation but also impedes the development of selective *O*‐GlcNAcylation‐targeting STING inhibitors. Therefore, further elucidation of the mechanisms underlying STING *O*‐GlcNAcylation and identification of small molecules that modulate this modification may facilitate the development of STING‐targeted immunomodulators.

Conventional STING inhibitors broadly suppress the overall biological activity of STING [17]. However, because STING participates in multiple physiological and pathological processes, systemic inhibition may disrupt beneficial STING‐dependent functions while also increasing the risk of off‐target effects [18]. This underscores the necessity for more precise targeting strategies that selectively modulate disease‐specific aberrant activation states of STING, rather than indiscriminately inhibiting the entire function of STING. Such precision interventions could inhibit pathogenic STING signaling while preserving its physiological functions, thereby enabling safer and more effective treatments for psoriasis and other immune‐mediated inflammatory diseases. Unfortunately, the majority of current drug development efforts, such as ligand‐competitive inhibitors (e.g., SN‐011) [19], polymerization inhibitors (e.g., LB244) [20], and degraders (e.g., AK59) [21], continue to focus on the global modulation of STING activity, thereby lacking the molecular precision to achieve selective therapeutic effects.

Paeoniflorin (PF), a bioactive terpenoid glycoside derived from *Paeonia lactiflora* Pall., a traditional Chinese medicinal herb widely used for rheumatic and immune‐related diseases, has demonstrated potent anti‐inflammatory efficacy across various immune disorders [22, 23]. In this study, we identify PF as an allosteric STING inhibitor that alleviates macrophage‐driven inflammatory responses in psoriasis by selectively suppressing STING *O*‐GlcNAcylation. Mechanistically, PF specifically binds to the cyclic dinucleotide‐binding domain (CBD) of STING and acts as a molecular “lock” to stabilize the protein in an inactive open conformation, with Thr229, a key *O*‐GlcNAcylation site, positioned within the PF‐binding interface. This conformational change impairs *O*‐GlcNAc transferase (OGT) recruitment, suppresses STING *O*‐GlcNAcylation, and consequently inhibits downstream STING phosphorylation and NF‐κB signaling. Functionally, genetic ablation of STING in macrophages reduces pro‐inflammatory chemokine secretion and ameliorates psoriatic pathology. Furthermore, analyses of human psoriasis samples revealed an association between macrophage‐associated STING *O*‐GlcNAcylation and psoriasis disease severity.

Collectively, our results identify PF as an allosteric inhibitor of STING and support targeting STING *O*‐GlcNAcylation in macrophages as a promising therapeutic strategy for psoriasis.

## 2 Results

### 2.1 PF Suppresses Macrophage‐Driven Pathogenic Phenotypes in Psoriasis

The pathogenesis of psoriasis is driven by the activation of multiple immune cells. To characterize the immune microenvironment of psoriatic skin, we analyzed a publicly available single‐cell RNA‐seq dataset from GEO (GSE173706) [24], comprising two peripheral normal skin samples from patients with psoriasis (GSM5277178, GSM5277181) and three psoriatic lesional skin samples (GSM5277189, GSM5277190, GSM5277191). Unsupervised clustering identified nine major cell populations, with macrophages exhibiting a marked increase in both absolute number and relative proportion in psoriatic lesions compared to non‐lesional skin, positioning them among the most significantly expanded immune cell types (Figure 1A; Figure S1A). To determine whether macrophage enrichment was recapitulated in experimental psoriasis‐like inflammation, we analyzed a publicly available mouse skin single‐cell RNA‐seq dataset from GEO (GSE149121) [25], comprising control and IMQ‐treated samples. Similarly, the proportion of macrophages among the profiled CD45^+^ immune cells was increased in IMQ‐induced psoriasis‐like skin compared with control skin (Figure S1B). These cross‐species single‐cell analyses indicate an important role for macrophages in psoriasis‐associated skin inflammation, consistent with previous studies implicating macrophages in psoriasis pathogenesis [26]. Immunofluorescence staining further showed increased CD14^+^ and CD68^+^ macrophage infiltration in the dermis of psoriatic lesions (Figure 1B). Cell–cell interaction analysis revealed enhanced communication between macrophages and fibroblasts, keratinocytes, and natural killer T cells, supporting a central role for macrophages in inflammatory crosstalk in psoriasis (Figure S1C).

**FIGURE 1:**
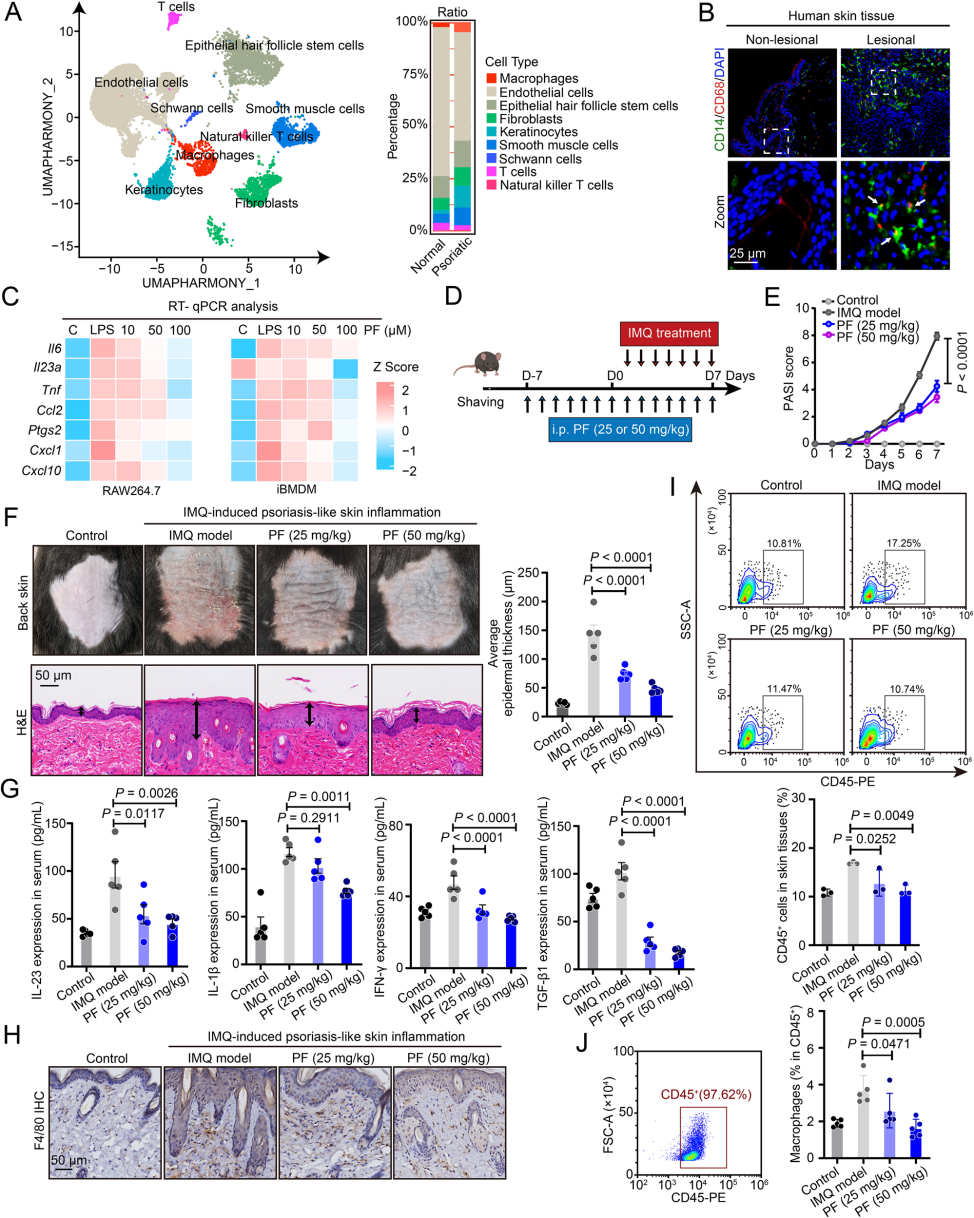
PF suppresses macrophage‐driven pathogenic phenotypes in psoriasis. (A) UMAP of scRNA‐seq data from two normal skin samples and three lesional skin samples from patients with psoriasis, showing annotated cell types and their relative proportions. Data were obtained from GEO dataset GSE173706 (normal: GSM5277178 and GSM5277181; psoriasis: GSM5277189, GSM5277190, and GSM5277191). (B) Representative images of CD14 (green) and CD68 (red) immunofluorescence staining in non‐lesional and lesional human skin tissues from patients with psoriasis (*n* = 3). Scale bars, 25 µm. (C) Heatmap of relative mRNA expression of the indicated genes in RAW264.7 cells or iBMDMs stimulated with LPS and treated with PF (0 µm, 10 µm, 50 µm, and 100 µm), as determined by RT‐qPCR. (D) Schematic of the PF pretreatment strategy in the IMQ‐induced psoriasis model. (E) PASI scores in mice with IMQ‐induced psoriasis treated with PF (*n* = 6). (F) Representative images of dorsal skin and H&E‐stained sections from mice with IMQ‐induced psoriasis treated with PF, with quantification of epidermal thickness (*n* = 5). Scale bars, 50 µm. (G) ELISA analysis of serum IL‐23, IL‐1β, IFN‐γ, and TGF‐β1 levels in mice with IMQ‐induced psoriasis treated with PF (*n* = 5). (H) IHC staining of F4/80 in lesional skin from mice with IMQ‐induced psoriasis treated with PF (*n* = 3). Scale bars, 50 µm. (I) Flow cytometry analysis of CD45^+^ immune cells infiltrating the skin of mice with IMQ‐induced psoriasis‐like inflammation treated with PF (*n* = 3). (J) Flow cytometry analysis of splenic macrophages among CD45^+^ leukocytes in mice with IMQ‐induced psoriasis‐like inflammation treated with PF (*n* = 5). Data are presented as mean ± SEM. Statistical significance was determined by one‐way ANOVA followed by Dunnett's multiple comparisons test.

These observations prompted us to examine whether paeoniflorin (PF) directly suppresses macrophage inflammatory responses. In LPS‐stimulated RAW264.7 cells and iBMDMs, PF concentration‐dependently reduced the mRNA expression of proinflammatory cytokines and chemokines, including *Il6*, *Il23a*, *Tnf*, *Ccl2*, *Ptgs2*, *Cxcl1*, and *Cxcl10* (Figure 1C). We also examined other psoriasis‐relevant cell types and found that PF treatment did not alter HaCaT cell viability (Figure S1D) or LPS‐induced *Il23a* expression and IL‐23 secretion in bone marrow‐derived dendritic cells (BMDCs) (Figure S1E,F). The anti‐inflammatory activity of PF was then evaluated in vivo. PF attenuated CuSO_4_‐induced acute inflammation in zebrafish (Figure S2A). In the IMQ‐induced psoriasis model, PF pretreatment significantly alleviated skin inflammation, as evidenced by reduced lesion severity, lower Psoriasis Area and Severity Index (PASI) scores, attenuated epidermal hyperplasia, and diminished scaling (Figure 1D–F). In addition, PF markedly reduced the IMQ‐induced elevation of psoriasis‐associated cytokines (IL‐23, IL‐1β, TGF‐β1, and IFN‐γ) (Figure 1G). Immunofluorescence staining showed reduced F4/80^+^ macrophage accumulation and CD45^+^ immune‐cell infiltration in lesional skin following PF treatment (Figure 1H,I). Flow cytometric analysis of splenocytes similarly revealed a significant reduction in CD11b^+^F4/80^+^ macrophages within the CD45^+^ immune cell population (Figure 1J; Figure S2B). Moreover, we examined the effects of PF in an IL‐23‐induced psoriasis‐like ear inflammation model, with methotrexate (MTX) included as a positive control. As shown in Figure S2C–E, PF treatment reduced ear swelling, epidermal hyperplasia, and epidermal thickness, showing effects comparable to those of MTX. Collectively, these data demonstrate that PF ameliorates psoriatic skin inflammation while attenuating macrophage activation and associated inflammatory responses.

### 2.2 STING Is a Cellular Target of PF in Macrophages

To systematically identify the direct molecular targets of PF in macrophages, we employed affinity‐based chemical proteomics approaches to identify PF‐binding proteins (Figure 2A; Figure S3A). Proteomics analysis revealed 1616 proteins visualized in a volcano plot (Figure 2B). Among these, 25 candidate targets met predefined criteria (light/heavy ratio >1.5, *p* < 0.05), including STING, TLR2, TLR7, and DHX9, which are functionally associated with inflammatory and immune regulation. To evaluate the contribution of these candidates to PF‐mediated suppression of macrophage activation, siRNA‐mediated knockdown of STING, TLR2, TLR7, or DHX9 was performed in RAW264.7 cells (Figure S3B–D,G). Importantly, PF‐induced inhibition of *Il6, Il23a, Ccl2*, and *Ptgs2* expression was attenuated in STING‐knockdown macrophages (Figure 2C), whereas silencing of TLR2, TLR7, or DHX9 did not compromise the anti‐inflammatory effects of PF (Figure S3E,F,H). These findings support STING as a key mediator of PF‐mediated inhibition of macrophage activation and associated inflammatory responses.

**FIGURE 2:**
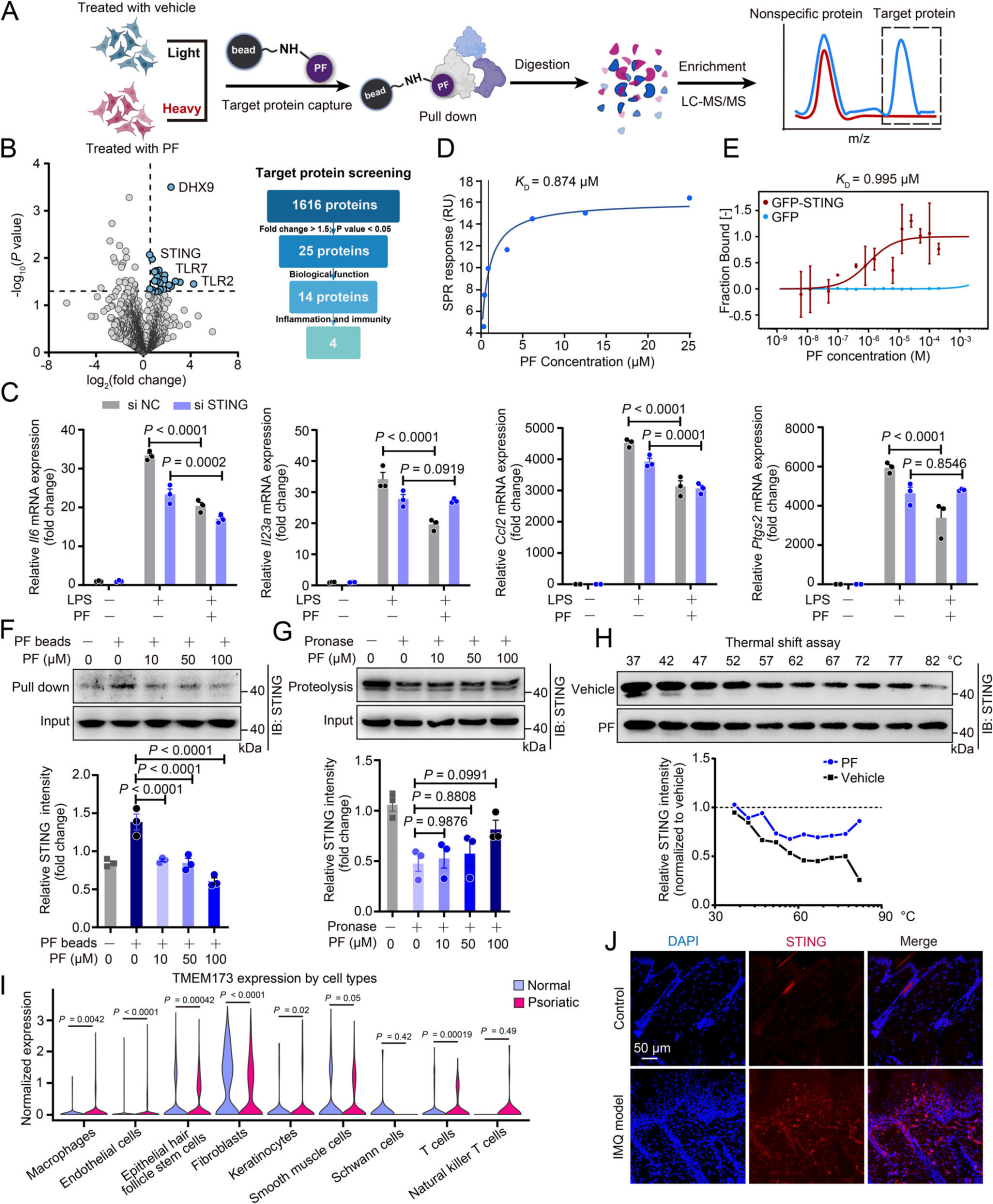
STING is a cellular target of PF in macrophages. (A) Schematic workflow for identifying PF‐binding targets by SILAC‐based PF‐bead pull‐down and LC–MS/MS analysis. (B) Volcano plot showing the protein targets of PF. The x‐ and y‐axes represent log_2_(fold change) and −log_10_(*p* value), respectively (*n* = 3). (C) RT‐qPCR analysis of *Il6*, *Il23a*, *Ccl2*, and *Ptgs2* mRNA levels in RAW264.7 cells transfected with STING siRNAs or NC siRNA, followed by LPS stimulation (1 µg/mL) with or without PF treatment (100 µM) (*n* = 3). Data are presented as mean ± SEM. Statistical significance was determined by two‐way ANOVA followed by Dunnett's multiple comparisons test. (D) SPR analysis of PF binding to STING. A representative result from three independent experiments is shown. (E) MST analysis of the interaction between PF and GFP‐STING (*n* = 3). (F) Pull‐down analysis of PF binding to STING with quantification (*n* = 3). (G) DARTS analysis of STING stability in the presence of PF with quantification (*n* = 3). (H) CETSA analysis of STING thermal stability in the presence of PF at the indicated temperatures (*n* = 3). (I) Violin plot of *TMEM173 (STING)* expression across different cell populations in peripheral normal and psoriatic human skin single‐cell RNA‐seq data (GSE173706). (J) Immunofluorescence analysis of STING (red) with DAPI counterstaining (blue) in skin sections from IMQ‐induced psoriatic mice (*n* = 3). Scale bars, 50 µm. Data are presented as mean ± SEM. Statistical significance was determined by one‐way ANOVA followed by Dunnett's multiple comparisons test unless otherwise indicated.

To validate STING as a direct target of PF, we next examined the interaction between PF and STING using multiple complementary approaches. SPR analysis revealed strong affinity between PF and STING, with a *K*_D_ value of 0.874 µm (Figure 2D), corroborated by an MST‐derived *K*_D_ of 0.995 µm (Figure 2E). Pull‐down assays using PF‐conjugated beads demonstrated specific binding of STING, which was effectively competed by excess free PF (Figure 2F). Additionally, drug affinity responsive target stability (DARTS) assays showed that PF protected endogenous STING from protease‐mediated degradation in both murine RAW264.7 and human THP‐1 cell lysates (Figure 2G; Figure S3I), while cellular thermal shift assays (CETSA) demonstrated enhanced STING thermostability upon PF treatment (Figure 2H). Having established the interaction between PF and STING, we then examined STING expression across major skin cell populations in both human and mouse single‐cell RNA‐seq datasets. STING (*TMEM173* in humans and *Tmem173* in mice) was detectable in multiple cell types, with relatively high expression in myeloid populations, particularly macrophages (Figure 2I; Figure S3J). Notably, STING expression in macrophages was significantly elevated in psoriatic skin lesions compared with healthy skin (Figure 2I). Consistent with this observation, immunofluorescence staining showed increased STING signals in macrophages in psoriatic‐like skin lesions from IMQ‐treated mice (Figure 2J). Taken together, these data identify macrophage STING as a functional target mediating the anti‐psoriatic effects of PF.

### 2.3 PF Allosterically Induces STING in an Inactive Open Conformation

To define the PF‐binding region on STING, we conducted a DARTS assay coupled with LC–MS/MS analysis. As shown in Figure 3A, peptides corresponding to residues 197–231 within the STING CBD were preferentially protected from proteolysis in the presence of PF, suggesting that PF engages a region within the CBD of STING. To further characterize the PF‐binding interface, molecular docking using the human STING (hSTING) crystal structure (PDB: 4KSY) predicted that PF forms hydrogen‐bond interactions with Glu174 (2.78 Å), Thr181 (2.89 Å), Gln227 (2.90 Å), and Thr229 (2.99 Å and 3.35 Å) (Figure 3B). To experimentally evaluate the contribution of these predicted residues to PF binding, we generated a GFP‐tagged hSTING‐4A mutant in which Glu174, Thr181, Gln227, and Thr229 were simultaneously substituted with alanine (E174A/T181A/Q227A/T229A). MST analysis showed that PF bound to hSTING‐WT with a *K_D_* value of 1.66 µM, whereas its binding affinity for hSTING‐4A was substantially decreased, with a *K_D_* value of 70.87 µM (Figure S4A,B). Docking analysis using mouse STING (PDB: 4KC0) similarly predicted PF binding within the CBD (Figure S5A). Together, these findings support the STING CBD as a PF‐binding region in both human and mouse STING.

**FIGURE 3:**
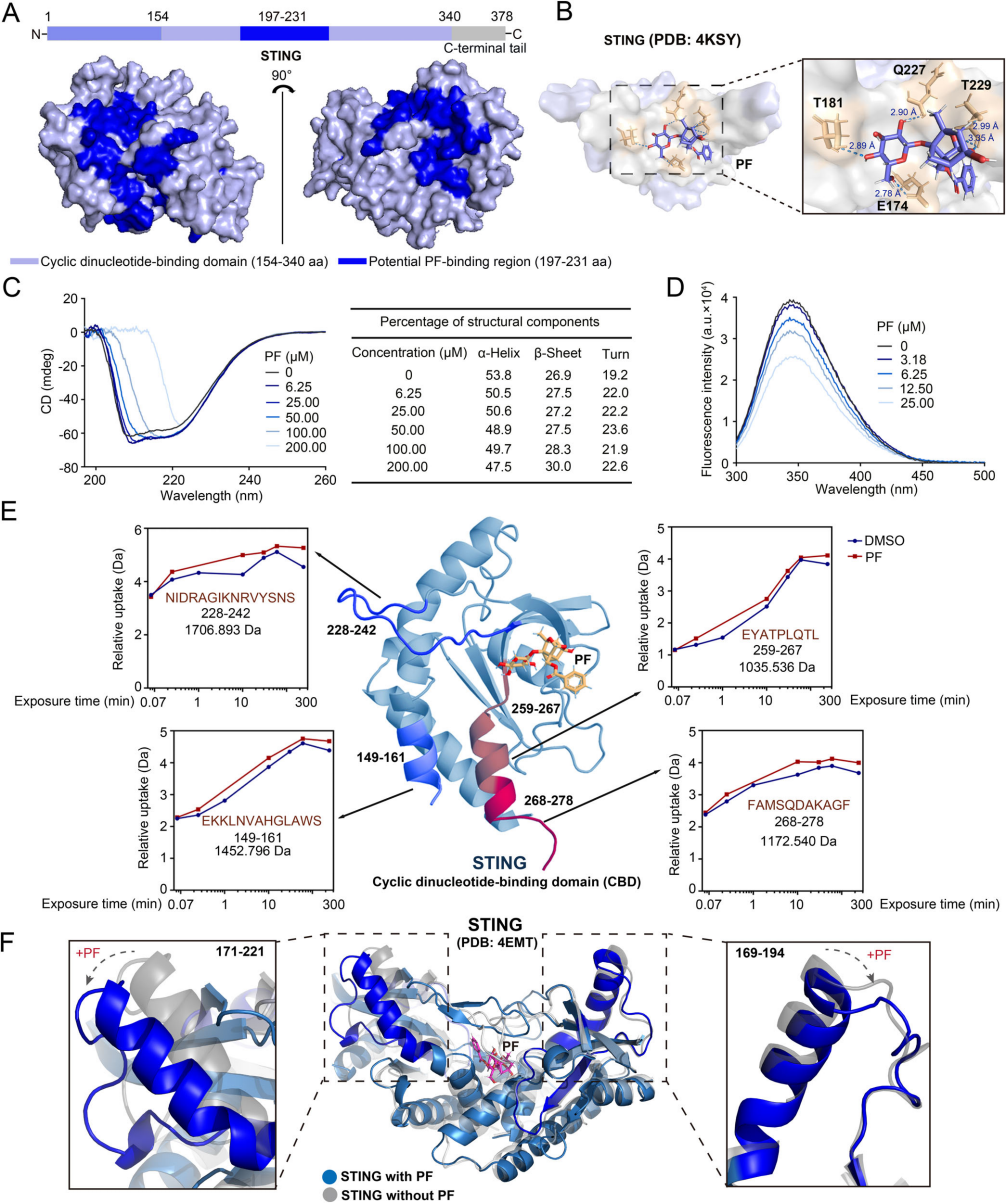
PF allosterically induces STING into an inactive open conformation. (A) Schematic illustration of the predicted PF‐binding region in STING based on DARTS‐MS analysis. (B) Molecular docking analysis of PF binding to human STING (PDB: 4KSY). (C) Circular dichroism (CD) spectroscopy analysis of STING. (D) Tryptophan fluorescence scanning analysis of STING. (E) Hydrogen‐deuterium exchange mass spectrometry (HDX‐MS) analysis of STING following PF treatment. Peptides exhibiting increased deuterium uptake were highlighted in color. (F) Representative structure of the PF‐bound human STING dimer (PDB: 4EMT) after 1 µs molecular dynamics simulation.

We next investigated the effect of PF binding on STING conformation. Circular dichroism (CD) spectroscopy analysis showed that PF treatment caused changes in the far‐UV CD spectrum of STING, particularly in the α‐helical region, indicative of altered secondary structure. Quantitative analysis revealed an overall reduction in α‐helix content accompanied by increases in β‐sheet and turn structures (Figure 3C). Complementary tryptophan fluorescence assays demonstrated concentration‐dependent fluorescence quenching upon PF treatment (Figure 3D), further supporting PF‐induced conformational changes in STING. Then, hydrogen‐deuterium exchange mass spectrometry (HDX‐MS) analysis revealed PF significantly increased deuterium incorporation in four discrete peptide segments (149–161, 228–242, 259–267, and 268–278) compared to untreated controls (Figure 3E). In contrast to STING agonists that induce an open‐to‐closed conformational transition upon CBD binding [27], molecular dynamics simulations showed that PF maintained STING in an open, inactive‐like conformation. Consistently, PF promoted an outward displacement of the 171–221 helical region, thereby favoring a more open conformation of the STING dimer (Figure 3F). PF binds the CBD of STING and allosterically stabilizes an inactive conformation, thereby preventing the rotational rearrangement and lid formation required for activation. We additionally performed molecular dynamics simulations using mouse STING (PDB: 4KC0) to assess the conservation of the allosteric effect of PF. The simulations showed that PF similarly stabilized an open conformation through the CBD region in mouse STING (Figure S5B). Overall, these analyses identify PF as an allosteric modulator of STING and provide a structural basis for the inhibitory effect on STING.

### 2.4 PF Significantly Suppresses STING *O*‐GlcNAcylation in Macrophages

STING activity is modulated by multiple PTMs, and conformational changes in STING could influence the accessibility of modification sites and the binding of PTM‐regulating enzymes [28, 29]. We therefore asked whether PF binding to the STING CBD could allosterically modulate STING post‐translational modifications and thereby regulate its activity. To investigate the underlying mechanism, we performed coimmunoprecipitation (co‐IP) coupled with mass spectrometry to identify STING‐associated proteins involved in the disease process. Among the identified proteins, OGT, the enzyme that catalyzes protein *O*‐GlcNAcylation by transferring GlcNAc from UDP‐GlcNAc to serine or threonine residues, was of particular interest (Figure 4A,B). Subsequent co‐IP assays demonstrated that PF treatment weakened the endogenous interaction between STING and OGT in iBMDMs (Figure 4C,D). Structural modeling further placed PF on STING near the predicted OGT‐binding region, supporting a model in which PF binding to STING hinders OGT association (Figure 4E). Consistent with this interpretation, MM/GBSA analysis suggested that PF weakened the predicted interaction between STING and OGT, as indicated by a shift in binding free energy from −93.28 to −85.18 kcal/mol (ΔΔG = +8.10 kcal/mol).

**FIGURE 4:**
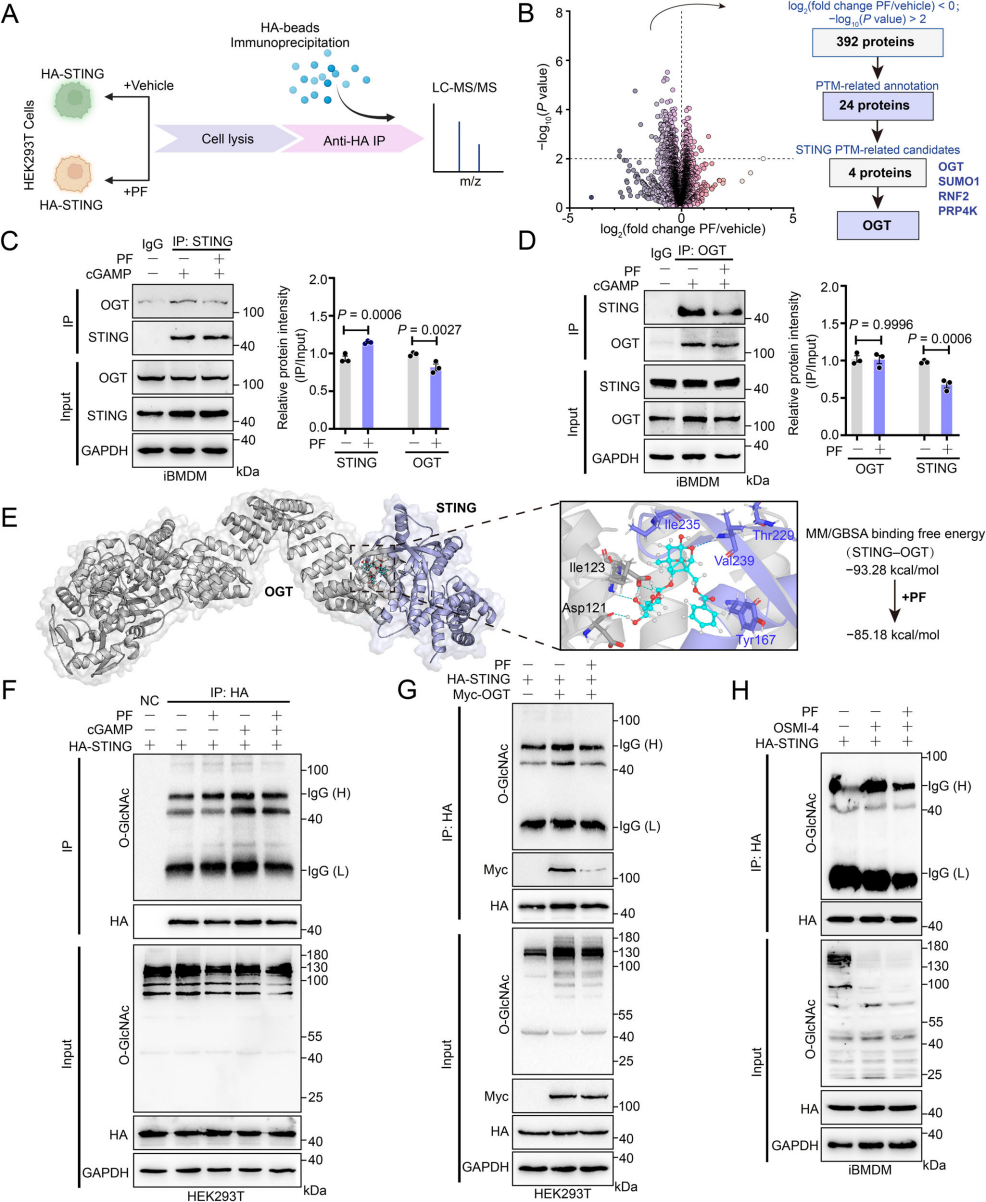
PF significantly suppresses STING *O*‐GlcNAcylation in macrophages. (A) Schematic diagram of the experimental design for co‐IP coupled with LC–MS/MS analysis. HEK293T cells were transfected with HA‐tagged STING, treated with PF (100 µm), and subjected to anti‐HA co‐IP followed by LC–MS/MS analysis of STING‐associated protein complexes (*n* = 3). (B) Screening workflow for STING‐interacting proteins. (C) Co‐IP analysis of the interaction between endogenous STING and OGT using an anti‐STING antibody. Band intensities were quantified by ImageJ (*n* =  3). (D) Co‐IP analysis of the interaction between endogenous OGT and STING using an anti‐OGT antibody. Band intensities were quantified by ImageJ (*n*  = 3). (E) Representative structure from molecular dynamics simulations of the OGT–STING complex in the presence of PF, with corresponding MM/GBSA binding free‐energy analysis in the absence or presence of PF. (F) HA immunoprecipitation from HEK293T cells treated with cGAMP (5 µm) and PF (100 µm), followed by immunoblotting with anti‐HA and anti‐O‐GlcNAc antibodies (*n*  = 3). NC, control beads without anti‐HA antibody. (G) HA immunoprecipitation from HEK293T cells co‐expressing HA‐STING and Myc‐OGT, followed by immunoblotting with anti‐HA, anti‐Myc, and anti‐*O*‐GlcNAc antibodies after PF (100 µm) treatment (*n*  = 3). (H) STING *O*‐GlcNAcylation was assessed by HA immunoprecipitation in iBMDMs transfected with HA‐STING and treated with OSMI‐4 (10 µm) or PF (100 µm) (*n*  = 3). Data are presented as mean ± SEM. Statistical significance was determined by one‐way ANOVA followed by Dunnett's multiple comparisons test.

Furthermore, immunoprecipitation followed by immunoblot analysis using a pan‐*O*‐GlcNAc antibody confirmed the *O*‐GlcNAcylation of STING, and PF treatment substantially reduced this modification (Figure 4F). Accordingly, overexpression of OGT enhanced STING *O*‐GlcNAcylation, which was effectively suppressed by PF (Figure 4G). Conversely, treatment with the OGT inhibitor OSMI‐4 substantially decreased STING *O*‐GlcNAcylation (Figure 4H). In addition, inhibition of *O*‐GlcNAcase (OGA) with Thiamet‐G increased STING *O*‐GlcNAcylation, which was only partially attenuated by PF treatment (Figure S6A). Of note, global cellular *O*‐GlcNAcylation levels remained unchanged upon PF treatment (Figure S6B), suggesting that PF does not broadly inhibit OGT‐mediated protein *O*‐GlcNAcylation. To address the possibility that PF directly engages OGT, we performed DARTS analysis by examining the protease susceptibility of endogenous OGT in the presence of PF. As shown in Figure S6C, PF did not confer detectable protection against proteolytic degradation, suggesting that PF does not appreciably bind OGT under these conditions. Thus, PF suppresses STING *O*‐GlcNAcylation by disrupting the STING–OGT interaction rather than by broadly inhibiting or directly engaging OGT.

Given the essential role of ER‐to‐Golgi trafficking in STING activation [30], we examined whether PF affects Golgi accumulation of STING during activation. Confocal microscopy indicated that PF partially attenuated cGAMP‐induced Golgi accumulation of STING, with a fraction of STING remaining outside the Golgi region (Figure S6D). In line with this observation, Cell Painting analysis revealed PF‐associated alterations in Golgi‐related phenotypic features (Figure S6E). Collectively, these data suggest that PF suppresses STING activation primarily by inhibiting OGT‐mediated STING *O*‐GlcNAcylation, accompanied by attenuated Golgi accumulation during activation.

### 2.5 STING *O*‐GlcNAcylation at Thr229 Modulates Distal Ser366 Phosphorylation

*O*‐GlcNAcylation has been reported to modulate phosphorylation [31, 32]. Thus, we asked whether PF‐mediated suppression of STING *O*‐GlcNAcylation occurs upstream of STING phosphorylation. To this end, we overexpressed OGT in iBMDMs and observed that elevated STING *O*‐GlcNAcylation was accompanied by enhanced STING phosphorylation. PF suppressed STING *O*‐GlcNAcylation and concomitantly reduced its phosphorylation (Figure 5A). In support of this, we generated OGT knockout iBMDMs using CRISPR‐Cas9 (Figure S7A) and found that OGT deficiency reduced STING *O*‐GlcNAcylation along with phosphorylation of STING and TBK1, without affecting STING protein stability (Figure 5B). These results indicate that the reduction in STING *O*‐GlcNAcylation contributes to the inhibitory effect of PF on STING phosphorylation.

**FIGURE 5:**
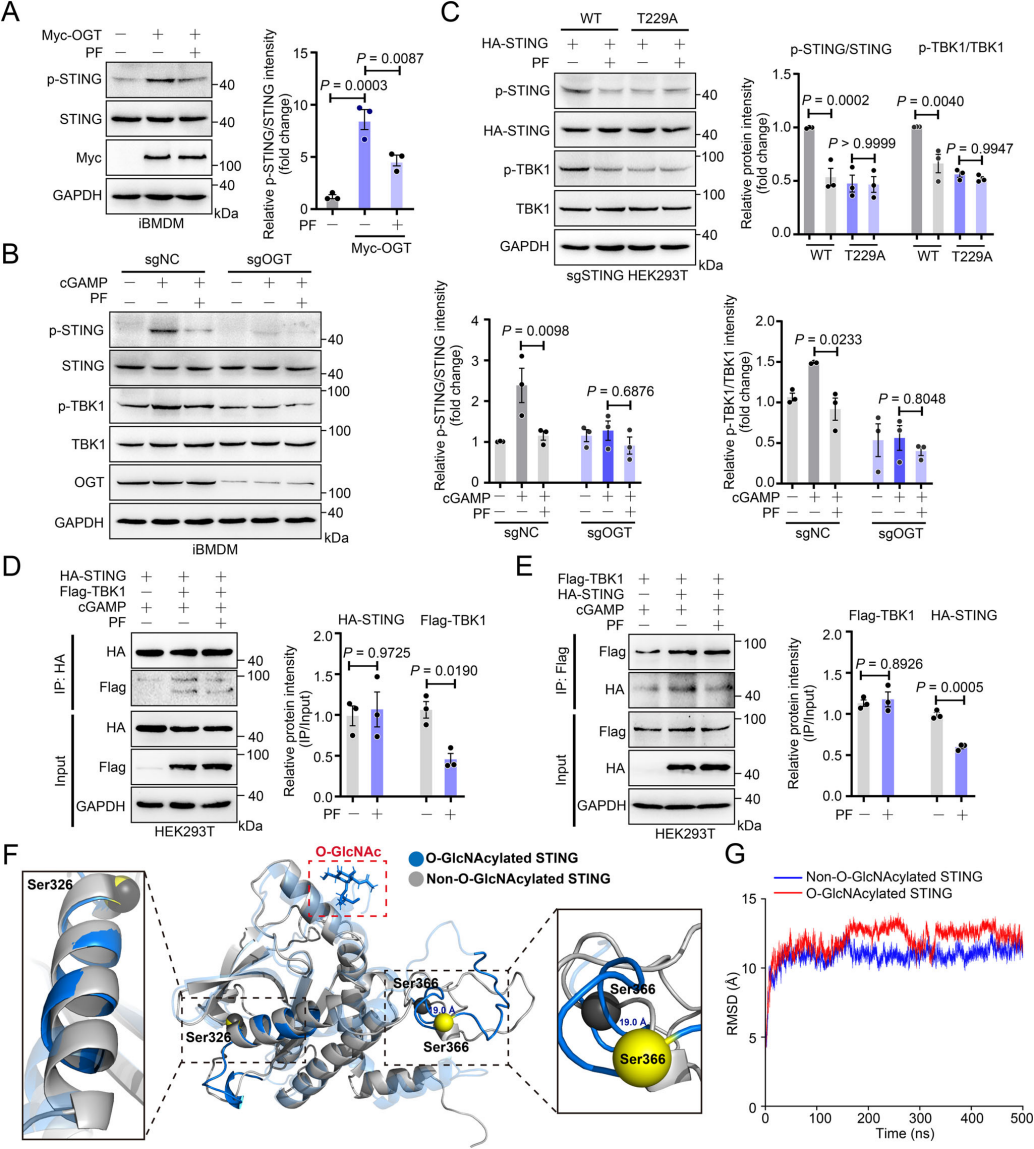
STING *O*‐GlcNAcylation at Thr229 modulates distal Ser366 phosphorylation. (A) Immunoblot analysis of iBMDMs transfected with Myc‐tagged OGT and treated with PF (100 µm). Band intensities were quantified by ImageJ (*n* = 3). (B) Immunoblot analysis of iBMDMs expressing sgNC or sgOGT and stimulated with cGAMP (5 µm) in the presence or absence of PF (100 µm). Band intensities were quantified by ImageJ (*n* = 3). (C) Immunoblot analysis of STING and TBK1 phosphorylation in STING‐deficient HEK293T cells reconstituted with WT or T229A STING and treated with PF (100 µm). Band intensities were quantified by ImageJ (*n* = 3). (D) HA immunoprecipitation analysis of the interaction between HA‐STING and Flag‐TBK1 in HEK293T cells treated with cGAMP (5 µm) in the presence or absence of PF (100 µm). Band intensities were quantified by ImageJ (*n* = 3). (E) Flag immunoprecipitation analysis of the interaction between Flag‐TBK1 and HA‐STING in HEK293T cells treated with cGAMP (5 µm) in the presence or absence of PF (100 µm). Band intensities were quantified by ImageJ (*n* = 3). (F) Representative conformations from 500 ns molecular dynamics simulations of Thr229 *O*‐GlcNAcylated and non‐*O*‐GlcNAcylated STING. (G) Backbone RMSD of non‐*O*‐GlcNAcylated and *O*‐GlcNAcylated STING during 500 ns molecular dynamics simulations. Data are presented as mean ± SEM. Statistical significance was determined by one‐way ANOVA followed by Dunnett's multiple comparisons test.

Thr229 has been reported as a potential *O*‐GlcNAcylation site on STING [16]. To confirm the functional significance of this residue, we generated STING‐knockout HEK293T cells (Figure S7B,C) and reintroduced either wild‐type STING (WT) or the T229A mutant (Figure S7D). Notably, mutation at Thr229 significantly attenuated STING phosphorylation due to impaired *O*‐GlcNAcylation (Figure 5C). Moreover, the inhibitory effect of PF on STING phosphorylation was lost in T229A mutant cells (Figure 5C), suggesting that PF primarily acts on *O*‐GlcNAcylation upstream of phosphorylation. As STING activation requires TBK1 recruitment and STING‐TBK1 complex formation in the ER‐Golgi intermediate compartment [33], we next examined whether PF affects this interaction. Co‐IP assays revealed that PF substantially disrupted the interaction between STING and TBK1, thereby impeding downstream STING signaling (Figure 5D,E). Based on these observations, we propose that *O*‐GlcNAcylation at Thr229 enhances STING activity by enabling spatial remodeling that facilitates downstream phosphorylation. Molecular dynamics simulations showed that Thr229‐linked *O*‐GlcNAc induces local structural shifts, displacing Ser366 in the flexible loop by ∼19 Å (Figure 5F) and leading to sustained conformational deviations, as evidenced by elevated RMSD over 500 ns (Figure 5G). Accordingly, PF binding to the STING CBD sterically blocks Thr229, preventing OGT‐mediated *O*‐GlcNAcylation and disrupting the spatial organization required for efficient phosphorylation. This is reflected in the altered positioning of the Ser366‐containing C‐terminal tail, which is important for downstream STING signaling (Figure 5F). In iBMDMs, cGAMP‐induced colocalization of *O*‐GlcNAc and phosphorylated STING was diminished by OSMI‐4 or PF (Figure S7E). Taken together, these results demonstrate that PF inhibits STING signaling primarily through steric hindrance, preventing Thr229 *O*‐GlcNAcylation and the consequent spatial remodeling necessary for downstream activation.

### 2.6 PF Attenuates STING‐Dependent TBK1‐NF‐κB Inflammation Signaling Activation

To determine whether PF modulates downstream signaling pathways following STING activation, we first conducted a STING‐driven IFNβ promoter luciferase reporter assay. Results demonstrated PF treatment significantly suppressed STING‐driven IFNβ promoter activity in a STING‐dependent manner (Figure 6A). Subsequent investigations revealed PF significantly attenuated phosphorylation of both STING and TBK1 following stimulation with the canonical STING agonist cGAMP (Figure 6B). Parallel experiments similarly showed PF inhibited LPS‐induced phosphorylation of these targets (Figure S8A). These findings were corroborated in vivo by immunohistochemical analysis of IMQ‐induced psoriatic lesions, where PF treatment reduced STING phosphorylation (Figure 6C). Importantly, gene expression pattern analysis revealed that the majority of cGAS–STING pathway‐related genes were upregulated in psoriatic lesions compared with normal skin (Figure 6D), indicating activation of cGAS–STING‐associated inflammatory signaling during psoriasis development. Thus, these findings identify PF as a STING‐targeting inhibitor with therapeutic potential in psoriasis.

**FIGURE 6:**
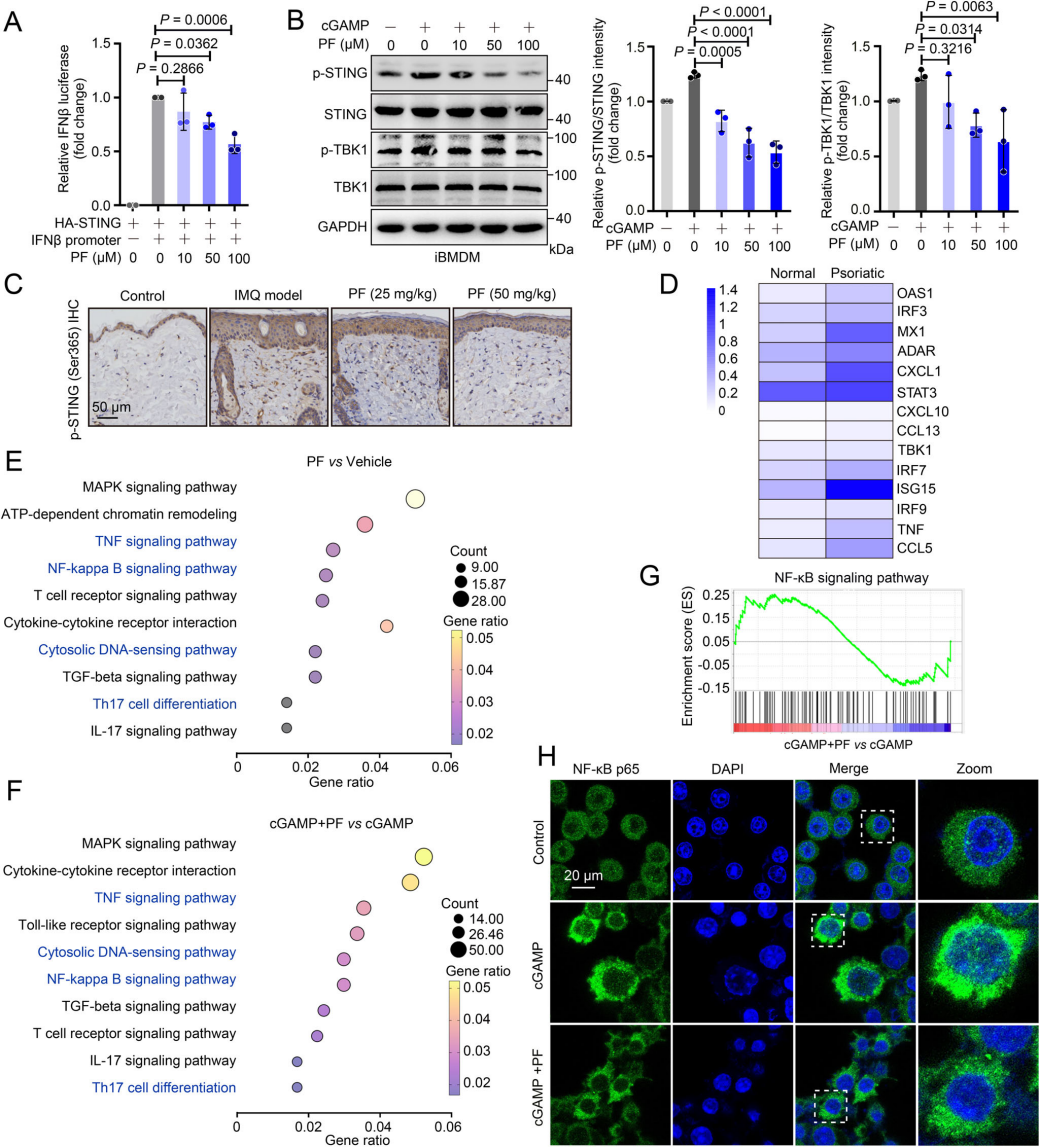
PF attenuates STING‐dependent TBK1‐NF‐κB inflammation signaling activation. (A) IFNβ promoter–driven luciferase assay of STING‐dependent reporter activity following PF treatment at the indicated concentrations (*n* = 3). (B) Immunoblot analysis of STING and TBK1 phosphorylation in iBMDMs treated with cGAMP (5 µm) and the indicated concentrations of PF. Band intensities were quantified by ImageJ (*n* = 3). (C) IHC analysis of p‐STING (Ser365) in skin sections from mice with IMQ‐induced psoriasis following PF treatment (*n* = 3). Scale bars, 50 µm. (D) Heatmap of cGAS–STING pathway‐related gene expression in human skin single‐cell RNA‐seq data from normal and psoriatic samples (GSE173706). (E) KEGG pathway enrichment analysis of RNA‐seq data from iBMDMs treated with PF (100 µm) or vehicle. (F) KEGG pathway enrichment analysis of RNA‐seq data from iBMDMs treated with cGAMP (5 µm) alone or in combination with PF (100 µm). (G) GSEA of RNA‐seq data from iBMDMs treated with cGAMP (5 µm) alone or in combination with PF (100 µm). (H) Representative immunofluorescence images of NF‐κB p65 nuclear localization in iBMDMs treated with cGAMP (5 µm) with or without PF (100 µm) (*n* = 3). Scale bars, 20 µm. Data are presented as mean ± SEM. Statistical significance was determined by one‐way ANOVA followed by Dunnett's multiple comparisons test.

To further characterize the effects of PF on downstream STING signaling, we profiled the transcriptional response to PF by RNA sequencing in the presence or absence of cGAMP (Figure S9A,B). Global transcriptomic analysis of macrophages showed that PF attenuated cGAMP‐induced transcriptional programs associated with the cytosolic DNA‐sensing, IL‐17, T cell receptor, and TNF signaling pathways (Figure 6E,F). Gene set enrichment analysis (GSEA) further revealed significant alterations in the NF‐κB and TNF signaling pathways following PF treatment (Figure 6G; Figure S8B). Previous evidence has shown that STING activation induces NF‐κB responses in myeloid cells [34], prompting us to examine whether PF attenuates STING‐associated NF‐κB activation. Western blot analysis showed that PF substantially reduced the phosphorylation of IKKβ, IκBα, and NF‐κB p65 following LPS stimulation in macrophages (Figure S9C). Immunofluorescence assay showed that nuclear translocation of the NF‐κB p65 subunit was enhanced by cGAMP or LPS stimulation, whereas PF treatment inhibited p65 translocation to nearly basal levels (Figure 6H; Figure S9D). In summary, our study provides evidence that PF significantly inhibits STING activation and TBK1 phosphorylation, thereby attenuating downstream NF‐κB signaling.

### 2.7 Macrophage‐Specific STING Deficiency Inhibits Psoriasis Progression in Mice

To investigate the role of STING in psoriasis progression, we generated myeloid‐specific STING knockout mice (Lyz2‐Cre^+/−^ Sting^fl/fl^, designated as cKO) by crossing Sting^fl/fl^ mice with Lyz2‐Cre mice (Figure 7A; Figure S10A,B). Littermates (Lyz2‐Cre^+/−^ Sting^+/+^) served as wild‐type controls (WT). Bone marrow‐derived macrophages (BMDMs) from cKO mice exhibited significantly reduced STING expression at both protein and mRNA levels compared to WT controls (Figure S10C,D). Multiplex immunofluorescence staining of lesional skin showed reduced STING signals in F4/80^+^ macrophages, Ly6G^+^ neutrophils, and Ly6C^+^ monocytes in cKO mice compared with WT mice (Figure S10E). Following IMQ application, myeloid STING deficiency ameliorated psoriatic phenotypes, including attenuated ear swelling and thickness, reduced epidermal hyperplasia, and lower PASI scores (Figure 7B,C). These data support a pathogenic role for myeloid STING in driving IMQ‐induced psoriasiform skin inflammation.

**FIGURE 7:**
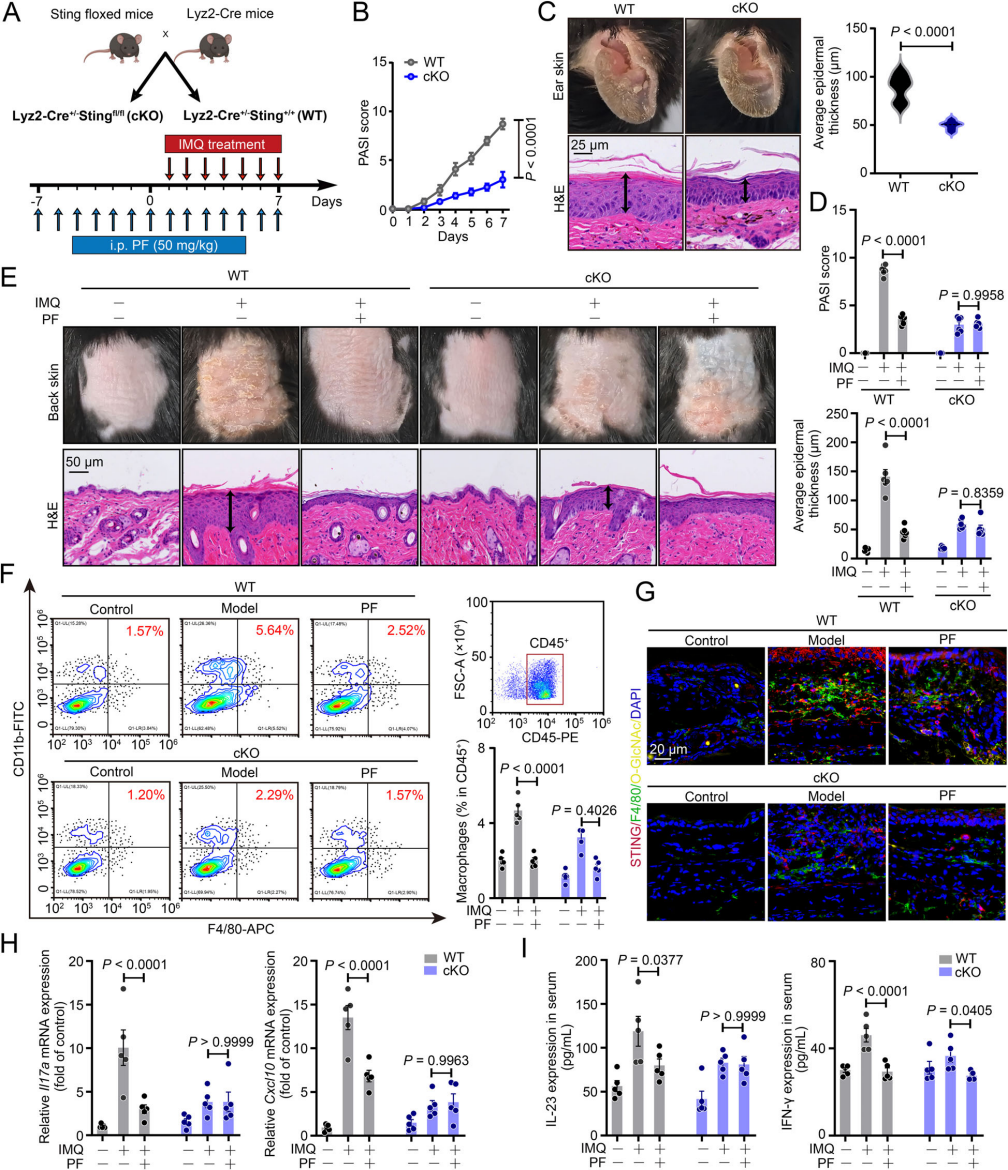
Macrophage‐specific STING deficiency inhibits psoriasis progression in mice. (A) Schematic illustration of myeloid‐specific STING cKO mouse generation and the experimental design of PF treatment in the IMQ‐induced psoriasis model. (B) Daily PASI scores in WT and STING cKO mice following topical application of IMQ or Vaseline control for 7 consecutive days (*n* = 6). (C) Representative images of ears and H&E‐stained skin sections from WT and STING cKO mice (*n* = 3). Scale bars, 25 µm. (D) PASI‐like scores of dorsal skin in WT and STING cKO mice on day 7 after IMQ induction, with or without PF treatment (*n* = 6). (E) Representative images of dorsal skin and H&E‐stained sections from WT and STING cKO mice after IMQ induction and PF treatment, with quantification of epidermal thickness (*n* = 6). Scale bars, 50 µm. (F) Flow cytometry analysis of macrophages among CD45^+^ cells isolated from spleens of WT and STING cKO mice after IMQ induction and PF treatment (*n* = 5). (G) Representative triple immunofluorescence staining of STING (red), F4/80 (green), and pan‐*O*‐GlcNAc (yellow) in ear tissues from WT and STING cKO mice in the indicated groups. Nuclei were counterstained with DAPI (blue) (*n* = 3). Scale bars, 20 µm. (H) RT‐qPCR analysis of the indicated genes in bone marrow cells isolated from WT and STING cKO mice after IMQ induction and PF treatment (*n* = 5). (I) ELISA analysis of serum IL‐23 and IFN‐γ levels after IMQ induction and PF treatment (*n* = 5). Data are presented as mean ± SEM. Statistical significance was determined by one‐way ANOVA followed by Dunnett's multiple comparisons test.

We next investigated macrophage responses to PF in vivo. As shown in Figure 7D,E, PF treatment reduced skin thickness and PASI scores in WT mice, whereas these effects were substantially attenuated in STING cKO mice. Flow cytometric analysis further showed that PF significantly reduced the IMQ‐induced increase in splenic CD11b^+^F4/80^+^ macrophages among CD45^+^ leukocytes in WT mice, whereas this effect was not significant in STING cKO mice (Figure 7F). These findings suggest that macrophage STING contributes to the therapeutic effects of PF. Given the association between STING *O*‐GlcNAcylation and STING‐driven inflammation in macrophages, we then assessed STING *O*‐GlcNAcylation in vivo. PF reduced STING *O*‐GlcNAcylation, an effect that was attenuated in STING cKO mice (Figure S10G). Triple immunofluorescence staining showed increased colocalization of STING and *O*‐GlcNAc signals within F4/80‐positive macrophage‐enriched regions in psoriatic skin. PF treatment reduced the STING/*O*‐GlcNAc overlap in WT mice, whereas this PF‐induced reduction was markedly attenuated in STING cKO mice (Figure 7G). PF also reduced IMQ‐induced inflammatory *Il17a* and *Cxcl10* expression (RT‐qPCR) as well as IL‐23 and IFN‐γ protein levels (ELISA) in WT mice, but not in STING cKO mice (Figure 7H,I). Collectively, these findings indicate that STING in macrophages plays an important role in the attenuation of psoriasis mediated by PF.

### 2.8 STING *O*‐GlcNAcylation Correlates with Disease Severity in Psoriasis Patients

To investigate the clinical significance of STING *O*‐GlcNAcylation in psoriasis pathogenesis, we performed bioinformatic analysis of single‐cell RNA‐sequencing datasets derived from human psoriatic lesions. Here, we systematically identified immune cell populations that co‐express STING and OGT, thereby elucidating the potential role in disease severity. UMAP‐based clustering showed that STING/OGT double‐positive cells were enriched primarily in macrophage, keratinocyte, fibroblast, and epithelial hair follicle stem cell populations (Figure 8A). In line with this distribution, STING transcripts were broadly increased in psoriatic lesions, whereas OGT expression was comparatively modest and heterogeneous across cell types (Figure 8B; Figure S11A), suggesting that STING *O*‐GlcNAcylation may occur in specific cellular subsets (such as macrophages) rather than reflecting a uniform lesion‐wide increase in OGT.

**FIGURE 8:**
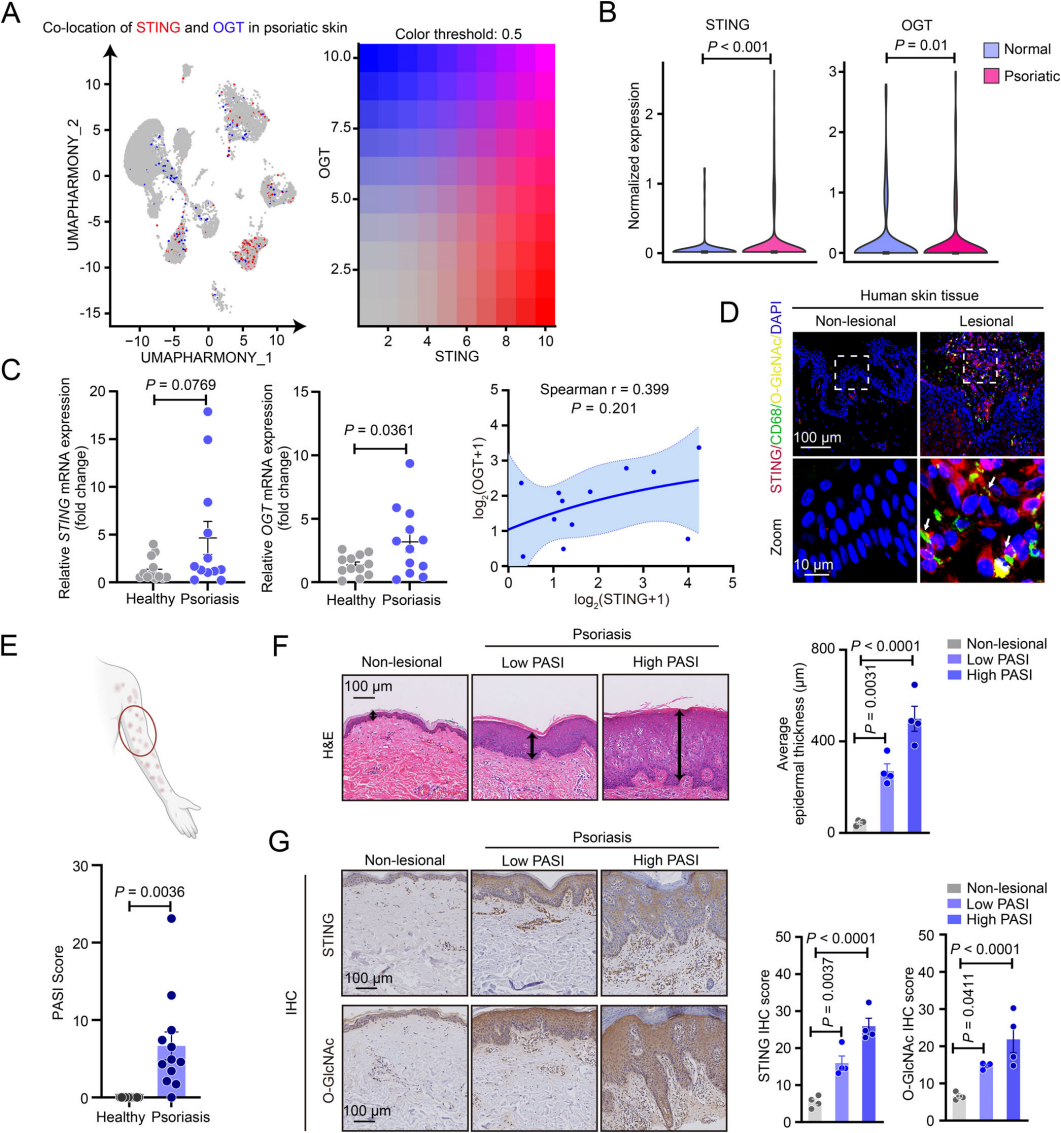
STING *O*‐GlcNAcylation correlates with disease severity in psoriasis patients. (A) UMAP visualization of STING and OGT expression in human skin single‐cell RNA‐seq data (GSE173706), with a bivariate color scale indicating their relative expression levels. (B) Violin plots of STING and OGT expression in human skin single‐cell RNA‐seq data (GSE173706). (C) RT‐qPCR analysis of *STING* and *OGT* mRNA expression in whole‐blood samples from healthy individuals and patients with psoriasis (n = 12), with Spearman correlation analysis in the psoriasis group. (D) Representative triple‐immunofluorescence images of STING (red), CD68 (green), pan‐*O*‐GlcNAc (yellow), and DAPI (blue) in non‐lesional and lesional skin sections from patients with psoriasis (*n* = 3). (E) PASI scores of the human psoriasis and healthy cohorts (*n* = 12). (F) Representative H&E staining of non‐lesional and lesional human skin sections from patients with psoriasis stratified by PASI score, with quantification of epidermal thickness (*n* = 4). Scale bars, 100 µm. (G) Representative IHC staining of STING and *O*‐GlcNAc in human skin sections from patients with psoriasis stratified by PASI score, with quantification of IHC scores (n = 4). Scale bars, 100 µm. Data are presented as mean ± SEM. Statistical significance was determined by one‐way ANOVA followed by Dunnett's multiple comparisons test unless otherwise indicated.

We next examined primary patient‐derived samples and observed elevated STING and OGT expression in psoriasis specimens relative to healthy controls (Figure 8C). Across these samples, STING and OGT levels showed a positive trend by Spearman correlation (*n* = 12; r = 0.399, *p* = 0.201) (Figure 8C). In parallel, analysis of peripheral blood samples from patients with psoriasis revealed elevated mRNA expression of inflammatory mediators, including *IFNB1, CXCL1, CXCL2, CXCL8, IL17A, IL23A*, and *TNF*, compared with healthy controls (Figure S11B,C). These data are consistent with an activated inflammatory state in psoriasis and provide further clinical evidence supporting the upregulation of STING and *O*‐GlcNAcylation. To validate this finding, triple immunofluorescence staining revealed relatively weak STING and pan‐*O*‐GlcNAc signals in non‐lesional skin, whereas lesional skin exhibited more prominent spatial overlap of these signals within CD68‐positive macrophages (Figure 8D). To assess the association of STING and *O*‐GlcNAc signaling with clinical disease severity, human skin specimens were stratified into low‐ and high‐PASI groups according to PASI scores and subjected to IHC staining for STING and global *O*‐GlcNAc (Figure 8E,F). Quantitative analysis indicated increased STING staining in high‐PASI lesions compared with low‐PASI lesions and non‐lesional skin (Figure 8G). Global *O*‐GlcNAc staining exhibited a similar pattern and positively correlated with PASI scores (Figure 8G). Together, these data suggest that enhanced STING expression and *O*‐GlcNAcylation are associated with psoriasis severity, supporting a potential link between STING‐related glycosylation signaling and disease progression.

## 3 Discussion

Pharmacological inhibition of STING represents a potentially attractive therapeutic strategy [35, 36]. However, the development of human STING antagonists remains limited [37], underscoring the need to investigate alternative modes of STING regulation. Here, we identify paeoniflorin (PF) as a STING‐targeting small molecule that suppresses macrophage‐driven psoriatic inflammation by interfering with STING *O*‐GlcNAcylation. Importantly, the anti‐psoriatic effects of PF were markedly attenuated in mice with myeloid‐specific deletion of STING, indicating that STING is a major functional target of PF in vivo. Our study reveals a previously unrecognized PTM‐dependent regulatory mechanism of STING activation and suggests PF as a potential chemical tool for modulating pathological STING signaling in inflammatory disease.

STING inhibition has traditionally focused on direct blockade of ligand binding or covalent targeting of residues required for activation. Beyond these established strategies, how conformational regulation intersects with post‐translational modifications to control STING activity remains incompletely understood. Unlike orthosteric antagonists that compete with cyclic dinucleotides at the canonical ligand‐binding pocket [19], or covalent inhibitors such as C‐176, C‐178, and H‐151 that target cysteine residues required for STING activation [38, 39], PF suppresses STING activation through a distinct non‐covalent conformational mechanism coupled to reduced STING *O*‐GlcNAcylation. Structure‐guided modeling and molecular dynamics simulations support a model in which PF binding to the STING CBD interferes with productive STING–OGT engagement, thereby reducing OGT‐mediated *O*‐GlcNAcylation at Thr229 and subsequent STING phosphorylation. The pharmacological modulation of other regulatory PTMs, such as STING palmitoylation, further suggests that post‐translational control represents a tractable layer of STING regulation [40]. Together with the recent identification of Y‐320 as an allosteric STING inhibitor [41], these findings highlight the pharmacological potential of targeting noncanonical regulatory sites in STING. Mechanistically, *O*‐GlcNAcylation may function as a dynamic regulatory modification that fine‐tunes STING activity in conjunction with its conformational state and interactions with signaling partners. *O*‐GlcNAcylated and non‐*O*‐GlcNAcylated forms of STING appear to differ in their capacity to undergo phosphorylation, oligomerization, intracellular trafficking, and downstream signal propagation. Thr229 *O*‐GlcNAcylation could favor an activation‐competent state of STING, whereas loss of this modification may shift STING toward a less signaling‐permissive state. Such dynamic regulation provides a potential mechanism by which *O*‐GlcNAcylation controls the magnitude and duration of STING‐dependent inflammatory signaling.

*O*‐GlcNAcylation is emerging as an important mechanism regulating STING activation and immune signaling. Recent studies have identified Thr229 *O*‐GlcNAcylation as a positive regulator of STING activation in antiviral innate immunity [16, 42]. Our findings extend the significance of STING *O*‐GlcNAcylation beyond antiviral defense by identifying its pro‐inflammatory role in psoriasis. Accumulating evidence indicates that aberrant glycosylation contributes to immune dysregulation in psoriasis and other autoimmune skin diseases, supporting glycosylation‐dependent signaling as a potential therapeutic target [43, 44]. Notably, the immunological effects of *O*‐GlcNAcylation are highly dependent on the modified protein and cellular context. For example, OGT‐mediated *O*‐GlcNAcylation of S6K1 can restrain pro‐inflammatory macrophage activation [45], whereas *O*‐GlcNAcylation of innate immune signaling proteins such as MAVS and IRF5 can enhance antiviral or inflammatory responses [46, 47]. These divergent effects highlight the importance of defining the functions of individual *O*‐GlcNAcylated proteins in distinct immune responses. STING‐dependent inflammation has also been implicated in psoriasis pathogenesis [48]. Our data indicate that increased STING *O*‐GlcNAcylation sustains STING activation in macrophages, whereas disruption of this modification reduces STING phosphorylation and downstream inflammatory signaling. Given the involvement of dysregulated STING signaling in multiple autoimmune and inflammatory disorders [49, 50], whether STING *O*‐GlcNAcylation similarly contributes to pathological inflammation in other disease settings remains to be determined.

*O‐*GlcNAcylation and phosphorylation can either oppose or reinforce each other depending on the protein and modification site [51]. In STING, Thr229 *O*‐GlcNAcylation and Ser366 phosphorylation occur at distinct residues [16, 52], suggesting that their crosstalk may involve mechanisms beyond direct competition at a single site. Several mechanisms could account for how *O*‐GlcNAcylation facilitates STING phosphorylation and signaling. One possibility is that Thr229 *O*‐GlcNAcylation within the STING CBD favors an activation‐competent conformation, thereby creating a structural state permissive for TBK1 recruitment and STING phosphorylation [53, 54]. Alternatively, *O*‐GlcNAcylation could promote or stabilize higher‐order STING assemblies, facilitating the organization of signaling complexes required for efficient phosphorylation and sustaining STING signaling over time [55, 56]. STING activation is also tightly linked to ER‐to‐Golgi trafficking [57, 58], raising the possibility that *O*‐GlcNAcylation influences the localization or persistence of activated STING within Golgi‐associated signaling compartments, thereby affecting its accessibility to TBK1. PF reduced cGAMP‐induced Golgi accumulation of STING in our experiments, indicating that PF also affects this trafficking‐dependent phase of STING activation; however, whether this effect is directly attributable to reduced Thr229 *O*‐GlcNAcylation remains to be determined. Because STING trafficking and turnover are important determinants of signal termination [59], changes in *O*‐GlcNAcylation could also influence the extent of STING phosphorylation and the duration of downstream inflammatory signaling. These mechanisms are not mutually exclusive and collectively provide possible explanations for how Thr229 *O*‐GlcNAcylation could facilitate efficient STING phosphorylation. However, the precise molecular basis linking Thr229 *O*‐GlcNAcylation to STING phosphorylation remains unresolved.

Despite these mechanistic insights, several limitations of the present study should be acknowledged. The concentrations of PF required to inhibit STING signaling in cells were higher than the low‐micromolar binding affinities measured in cell‐free assays. This discrepancy may reflect limited cellular uptake, transporter‐mediated efflux, intracellular distribution, and restricted accessibility of STING within membrane‐associated compartments. PF exposure in skin tissue and relevant immune‐cell populations was not measured, and whether comparable pharmacologically active concentrations are achieved at the relevant target sites in vivo remains unclear. The relatively small clinical cohort (*n* = 12) limits the statistical power and generalizability of the clinical observations, and larger independent cohorts will be needed to determine whether STING *O*‐GlcNAcylation is associated with disease severity. In addition, while STING‐specific *O*‐GlcNAcylation was supported by biochemical evidence from experimental models, direct biochemical validation in human psoriatic skin is still needed. Moreover, although biochemical and mutational analyses in experimental models support the involvement of Thr229 in STING *O*‐GlcNAcylation, direct site‐specific mass spectrometric confirmation and quantitative determination of its modification stoichiometry were not achieved. Our genetic and cellular data indicate an important contribution of macrophage STING to the effects of PF, but contributions from STING signaling in other cell types cannot be excluded. Finally, the structural basis linking PF binding to STING conformational changes and site‐specific *O*‐GlcNAcylation remains to be defined.

Collectively, our results identify STING *O*‐GlcNAcylation as a critical pathogenic mechanism in psoriasis and characterize PF as a STING allosteric inhibitor with a unique mode of action. This work also reveals promising therapeutic opportunities for psoriasis by targeting pathological STING activation.

## 4 Experimental Section

### 4.1 Chemicals and Reagents

PF and other major chemicals and reagents used in this study were obtained from the indicated commercial sources. Detailed information is provided in Table S1.

### 4.2 Cell Culture

Murine macrophage cells (RAW264.7), human embryonic kidney 293T cells (HEK293T), and human keratinocyte cells (HaCaT) were purchased from the Peking Union Medical College Cell Bank (Beijing, China). Human monocytic THP‐1 cells were purchased from Procell Life Science & Technology Co., Ltd. (Wuhan, China). Immortalized mouse bone marrow‐derived macrophages (iBMDMs) were generously provided by Prof. Fuping You's laboratory at the School of Basic Medical Sciences, Peking University. All cells were maintained in high glucose DMEM (except THP‐1 cells, which were maintained in RPMI 1640 medium) supplemented with 10% fetal bovine serum, 100 U/mL penicillin and 100 µg/mL streptomycin at 37°C in a 5% CO_2_ and 95% air humidified incubator.

### 4.3 Mice

Lyz2‐Cre and Sting^fl/fl^ mice on the C57BL/6J background were obtained from Shulaibao Biotechnology (Wuhan, Hubei, China). Myeloid‐specific STING conditional knockout mice (Lyz2‐Cre^+/−^ Sting^fl/fl^) were generated by crossing Sting^fl/fl^ mice with Lyz2‐Cre mice. The F1 generation carrying Lyz2‐Cre^+/−^ Sting^fl/+^ was used for further breeding. Among the Cre‐positive offspring, three genotypes were obtained: Lyz2‐Cre^+/−^ Sting^fl/fl^ (cKO), Lyz2‐Cre^+/−^ Sting^fl/+^ (Het) and Lyz2‐Cre^+/−^ Sting^+/+^ (WT). Genotypes were determined by polymerase chain reaction (PCR) analysis of genomic DNA isolated from toe biopsies, followed by agarose gel electrophoresis. The primer sequences used for mouse genotyping are listed in Table S2. All mice used in this study were 10‐week‐old male mice and were randomly assigned to experimental groups. All animal procedures were reviewed and approved by the Institutional Animal Care and Use Committee (IACUC) of Peking University Health Science Center (Approval No. DLASBD0747) and were conducted in accordance with the ARRIVE guidelines.

### 4.4 IMQ‐Induced Psoriasis‐Like Model

Imiquimod (IMQ) cream (5%; H20030128) was purchased from Sichuan Mingxin Pharmaceutical (Chengdu, Sichuan, China). Mice were shaved 7 days before IMQ application. PF was administered intraperitoneally once daily as a pretreatment for 7 days before IMQ application and then continued during IMQ treatment for an additional 7 days at the indicated doses (25 or 50 mg/kg). Psoriasis‐like dermatitis was induced by daily topical application of 62.5 mg of 5% IMQ cream to the shaved dorsal skin and ear for 7 consecutive days. Control mice received an equal amount of vehicle cream. Disease severity was assessed daily using a PASI‐like scoring system as previously described [60]. Erythema, scaling, and skin thickening were each scored on a scale from 0 to 4, and the cumulative score ranged from 0 to 12. Scoring was independently performed by two investigators blinded to the treatment groups, and the mean of the two scores was used for analysis. After the final IMQ application, dorsal skin and ear tissues were collected for H&E staining and immunohistochemistry (IHC). Mouse serum was isolated from whole blood by centrifugation and stored at −80°C until analysis.

### 4.5 Skin Cell Isolation and Flow Cytometry Analysis

Skin tissues from PF‐treated psoriatic model mice were incubated with dispase II (Sigma‐Aldrich, St. Louis, MO, USA) in Hanks’ Balanced Salt Solution at 37°C for 90 min to separate the epidermis and dermis. Dermal tissues were subsequently digested using a Skin Dissociation Kit (Bestopcell, Beijing, China) at 37°C for 60 min to generate single‐cell suspensions. Cells were stained with anti‐CD45–PE antibody from BD Biosciences (San Jose, CA, USA) and analyzed using a CytoFLEX flow cytometer from Beckman Coulter (Brea, CA, USA). Data were processed using CytExpert software from Beckman Coulter (Brea, CA, USA).

### 4.6 RNA Extraction and Real‐Time PCR Analysis

Cells were stimulated with lipopolysaccharide (LPS; 1 µg/mL) for 6 h in the presence or absence of PF (10 µm, 50 µm, or 100 µm). Total RNA was extracted using the MolPure Cell/Tissue Total RNA Kit (Yeasen, Shanghai, China) and reverse‐transcribed into cDNA using Hifair III first Strand cDNA Synthesis SuperMix (Yeasen, Shanghai, China). The resulting cDNA was diluted fivefold and subjected to quantitative real‐time PCR using Hieff qPCR SYBR Green Master Mix (Yeasen, Shanghai, China). Relative gene expression levels were calculated using the 2^(‐ΔΔCt)^ method with GAPDH as the internal control. Primer sequences used for real‐time PCR are listed in Table S3.

### 4.7 Preparation of PF‐Conjugated Amino‐Activated Agarose Beads (PF Beads)

An oriented immobilization strategy was employed to covalently conjugate the small molecule PF to amino‐activated agarose beads. Briefly, silicon dioxide (SiO_2_, 1.2 g) was added to dichloromethane (DCM, 100 mL) and stirred for 1 h. Sodium periodate (NaIO_4_, 28 mg) was then introduced, and the mixture was stirred thoroughly. PF (50 mg) was subsequently added, and the reaction was allowed to proceed for 24 h with continuous stirring. During this period, the vicinal diol groups of PF were selectively oxidized by NaIO_4_, generating reactive aldehyde groups. After completion of the oxidation reaction, the resulting intermediate was dissolved in toluene (30 mL). Amino‐activated Magrose beads (BeaverBeads Magrose NH_2_, 5 mg) were added, and the mixture was refluxed in toluene with stirring for 12 h, allowing the aldehyde groups of PF to react with the surface amino groups of the beads, thereby yielding PF‐conjugated beads.

### 4.8 Pull‐Down and LC–MS/MS Analysis for Target Identification

SILAC‐based pull‐down assays were performed in RAW264.7 cells. Cells were cultured in light or heavy SILAC medium for at least six passages to achieve > 95% labeling efficiency and then stimulated with LPS (1 µg/mL) for 1 h. Lysates were incubated with PF‐conjugated beads for 6 h at 4°C, with free PF (100 µm) added to the heavy‐labeled lysates for competition assays. Bead‐bound proteins were eluted, digested with trypsin, and analyzed by LC–MS/MS using an LTQ Velos Pro mass spectrometer (Thermo Fisher Scientific). Data were processed using Proteome Discoverer and searched with the SEQUEST algorithm at a peptide‐spectrum match FDR of 1%. Detailed sample preparation, MS acquisition, and database‐search parameters are provided in the Supporting Information.

### 4.9 Surface Plasmon Resonance (SPR) Assay

SPR experiments were performed using a Biacore 8K system (GE Healthcare, Boston, MA, USA) to quantify the binding affinity of PF for recombinant mouse STING (residues 149–378). Recombinant protein was immobilized on a CM5 sensor chip via standard amine coupling. PF, diluted in running buffer (50 mm Tris‐HCl, pH 7.2, 100 mm KCl), was injected at concentrations ranging from 0.39 µm to 100 µm. Binding data were analyzed using Biacore Insight Evaluation Software to determine the equilibrium dissociation constant (*K*_D_).

### 4.10 Hydrogen–Deuterium Exchange Mass Spectrometry (HDX–MS) Analysis

Recombinant mouse STING (residues 149–378; 1 mg/mL) was incubated with PF (100 µm) or vehicle at 4°C for 1 h. Hydrogen–deuterium exchange was initiated by diluting samples into deuterated labeling buffer (100 mm phosphate, D_2_O, pD 7.0) and allowed to proceed for various time points (5 s to 4 h). Exchange reactions were quenched with ice‐cold quenching buffer (100 mm phosphate, pH 2.0) and samples were immediately frozen in liquid nitrogen. Online proteolysis was performed using a Waters ENZYMATE BEH pepsin column (2.1 × 30 mm, 5 µm), followed by peptide trapping on a VanGuard Pre‐Column (ACQUITY UPLC BEH C18, 1.7 µm) and separation on an ACQUITY UPLC BEH C18 column (1.0 × 100 mm, 1.7 µm). Peptides were eluted at a flow rate of 100 µL/min with 15% acetonitrile. Mass analysis was performed on an Orbitrap Fusion Lumos mass spectrometer (Thermo Fisher Scientific, Waltham, MA, USA). Deuterium uptake was calculated by comparing the mass differences between deuterium‐labeled samples and non‐deuterated controls.

### 4.11 Immunofluorescence (IF) Assay

Cells were seeded onto confocal dishes and stimulated with the indicated inducer for the indicated time, followed by fixation with 4% paraformaldehyde for 30 min, permeabilization with 0.5% Triton X‐100 for 20 min, and blocking with 5% bovine serum albumin (BSA) for 30 min. Cells were incubated with primary antibodies (1:200) overnight at 4°C, followed by incubation with Alexa Fluor 488‐ or 546‐conjugated secondary antibodies for 1 h at room temperature. Nuclei were counterstained with DAPI or Hoechst 33342 for 20 min, and images were acquired using a Zeiss LSM 880 confocal microscope (Zeiss, Oberkochen, Germany). Alexa Fluor 488 (excitation, 488 nm; detection, 500–550 nm), Alexa Fluor 546 (excitation, 561 nm; detection, 570–620 nm), and DAPI or Hoechst 33342 (excitation, 405 nm; detection, 420–480 nm) were used for imaging.

### 4.12 Immunoprecipitation (IP) Analysis

Cells were lysed in IP buffer (50 mm Tris‐HCl, pH 7.4, 150 mm NaCl, 0.5% NP‐40, 1 mm EDTA, 10% glycerol) supplemented with a protease inhibitor cocktail at 4°C. Lysates were clarified by centrifugation at 12,000 g for 10 min at 4°C. Lysates were incubated with HA‐ or Flag‐conjugated beads overnight at 4°C. After five washes with ice‐cold lysis buffer, bound proteins were eluted with SDS‐PAGE loading buffer, boiled at 98°C for 10 min, and analyzed by immunoblotting.

### 4.13 IFNβ Promoter‐Luciferase Reporter Assay

HEK293T cells were seeded in 96‐well plates at 1.0 × 10^4^ cells/well 24 h before transfection. Cells were co‐transfected with IFNβ‐pGL3 firefly luciferase reporter plasmid (Addgene #102597; Watertown, MA, USA) and the indicated HA‐STING plasmids using PEI. DNA and PEI were separately diluted in Opti‐MEM, mixed for 15 min at room temperature, and added dropwise to cells. At 24 h post‐transfection, cells were treated with PF or vehicle as indicated, washed once with PBS, and lysed with 50 µL/well lysis buffer for 10 min at 600 rpm. Lysates were transferred to white opaque 96‐well plates, mixed with 50 µL/well luciferase working reagent, incubated for 5 min at room temperature, and measured using an INFINITE E PLEX microplate luminometer (Tecan, Männedorf, Zürich, Switzerland).

### 4.14 Genome Editing with CRISPR‐Cas9

Gene knockout cell lines were generated using the lentiCRISPRv2 system (Addgene plasmid #52961) as previously described [61]. Briefly, sgRNA oligonucleotides were annealed and ligated into Esp3I‐digested lentiCRISPRv2. Lentiviral particles were produced in HEK293T cells by co‐transfecting lentiCRISPRv2‐sgRNA with psPAX2 and pMD2.G, and the medium was replaced 6 h post‐transfection. Viral supernatants were collected at 48 h and 72 h post‐transfection, filtered through a 0.45 µm filter, and used to transduce target cells in the presence of polybrene (10 µg/mL). Medium was replaced 24 h after transduction, and cells were selected with puromycin (2 µg/mL) for 3 days. Single‐cell clones were obtained by limiting dilution and expanded. Knockout efficiency was validated by immunoblotting and/or Sanger sequencing of the targeted genomic locus. sgRNA sequences are listed in Table S4.

### 4.15 RNA Interference

Small interfering RNAs (siRNAs) targeting STING, TLR2, TLR7 and DHX9, together with a negative control siRNA, were transfected into RAW264.7 cells using Lipofectamine RNAiMAX (Thermo Fisher Scientific, Waltham, MA, USA). Briefly, siRNA and RNAiMAX were diluted separately in Opti‐MEM, combined, and incubated for 10–15 min at room temperature before being added to cells. At 24 h post‐transfection, cells were treated with PF (100 µm) for an additional 24 h. Protein expression levels of the indicated targets were analyzed by Western blotting. siRNA sequences are listed in Table S5.

### 4.16 Western Blotting

Proteins were separated by 10% SDS‐PAGE and transferred to PVDF membranes. Membranes were blocked with 5% (w/v) skim milk at room temperature for 30 min and incubated with primary antibodies overnight at 4°C. After washing with Tris‐buffered saline containing Tween‐20 (TBST), membranes were incubated with appropriate secondary antibodies for 1 h at room temperature. Protein bands were detected using enhanced chemiluminescence (ECL) and visualized with a Tanon 5200 Imaging Analysis System (Tanon, Shanghai, China). Detailed information on the antibodies used, including their sources, catalog numbers, applications, and working dilutions, is provided in Table S6.

### 4.17 Human Clinical Specimens

Skin biopsies were obtained from lesional and adjacent non‐lesional skin of patients with psoriasis vulgaris, while peripheral whole‐blood samples were collected from patients and healthy donors. The study was approved by the Ethics Committee of Guangdong Provincial Hospital of Chinese Medicine (Approval No. BF2021‐236‐01), and written informed consent was obtained from all participants prior to sample collection. All participants were enrolled voluntarily and received no financial compensation. Peripheral whole‐blood samples were subjected to RT‐qPCR analysis, and skin biopsy tissues were used for hematoxylin and eosin (H&E) staining and immunohistochemistry (IHC).

### 4.18 Statistical Analysis

All experiments were performed at least three times independently. Statistical analyses were performed using GraphPad Prism 11.0 software. Data are presented as the mean ± standard error of the mean (SEM). Comparisons between two groups were performed using an unpaired two‐tailed Student's *t*‐test. For comparisons among multiple groups, one‐way analysis of variance (ANOVA) followed by Dunnett's multiple comparisons test was used. For experiments involving two independent variables, two‐way ANOVA followed by an appropriate multiple comparisons test was performed. A *p* value < 0.05 was considered statistically significant.

## Author Contributions

F.‐F.Z. performed the majority of experiments, analyzed the data, and prepared the figures. C.‐C.H. contributed to key experimental validation, data analysis, and figure preparation. W.W., Y.‐X.Z., and L.L. assisted with molecular and cellular experiments, animal studies, and data analysis. L.‐J.L., C.‐J.L., and R.‐Z.Z. contributed to the collection of human psoriasis samples and clinical information. P.‐F.T. provided conceptual input and resources. Y.L., R.‐Z.Z., and K.‐W.Z. conceived and supervised the study, provided resources, and contributed to data interpretation. F.‐F.Z., Y.L., and K.‐W.Z. wrote and revised the manuscript. All authors reviewed and approved the final manuscript.

## Conflicts of Interest

The authors declare no conflicts of interest.

## Supporting information




**Supporting File**: advs78257‐sup‐0001‐SuppMat.docx.

## Data Availability

The data supporting the findings of this study are available within the article and its Supporting Information. The publicly available datasets analyzed in this study were obtained from GEO under accession numbers GSE173706 and GSE149121.
