# Supplementary material for: Targeting Macrophage STING O‐GlcNAcylation via Small‐Molecule Allosteric Modulation for Psoriasis Therapy
Source: Adv Sci (Weinh). 2026 Oct 14:e78257. Online ahead of print. doi: 10.1002/advs.78257 (PMC13669215; doi:10.1002/advs.78257)
Supplement: Supplementary file 1 [file ADVS-9999-e78257-s001.docx]

**^Supporting Information^**

**Targeting Macrophage STING *O*-GlcNAcylation via Small-Molecule Allosteric Modulation for Psoriasis Therapy**

Fang-Fang Zhuo^1^ ^†^, Cheng-Chen Hou^1^ ^†^, Wei Wang^1^, Ya-Xuan Zhu^1^, Ling Li^1^, Li-Juan Liu^2^, Chuan-Jian Lu^2^, Peng-Fei Tu^1^, Yang Liu^3*^, Rui-Zhi Zhao^2*^, Ke-Wu Zeng^1,4,5*^

^1^State Key Laboratory of Natural and Biomimetic Drugs, School of Pharmaceutical Sciences, Peking University, Beijing 100191, China.

^2^State Key Laboratory of Dampness Syndrome of Chinese Medicine, The Second Affiliated Hospital of Guangzhou University of Chinese Medicine, Guangzhou 510006, China.

^3^Center of Basic Medical Research, Institute of Medical Innovation and Research, Peking University Third Hospital, Beijing 100191, China.

^4^Department of Integration of Chinese and Western Medicine, School of Basic Medical Sciences, Peking University, Beijing 100191, China.

^5^Department of Integrated Traditional Chinese and Western Medicine, Peking University First Hospital, Beijing 100034, China.

†These authors contributed equally to this work.

*Corresponding author: [yliu_pu3h@bjmu.edu.cn](mailto:yliu_pu3h@bjmu.edu.cn) (Y Liu); zhaoruizhi@gzcm.edu.cn (RZ Zhao); ZKW@bjmu.edu.cn (KW Zeng)

**Method details**

**Protein expression and purification**

DNA fragments encoding His-tagged mouse STING (residues 149–378) were cloned into a modified pET-28a vector using the *NdeI* and *XhoI* restriction sites. DNA fragments encoding His-tagged human STING (residues 155–341) were cloned into a modified pQlinkGx vector using the *BamHI* and *EcoRI* restriction sites. Plasmid construction and sequence verification were performed by Ruibiotech (Beijing, China). The resulting constructs were transformed into *Escherichia coli* BL21 (DE3) cells (Solarbio, Beijing, China). Bacterial cultures were grown in LB medium at 37 °C with shaking at 220 rpm to an OD_600_ of ~0.6 and induced with 0.5 mM isopropyl β-D-thiogalactopyranoside (IPTG) at 16 °C for 24 h. Cells were harvested and lysed in buffer containing 20 mM HEPES (pH 7.5), 250 mM NaCl, 10 mM imidazole, and 10% glycerol. Recombinant proteins were purified by Ni–NTA affinity chromatography (GE Healthcare, Chicago, IL, USA) using a linear imidazole gradient (10–250 mM) in HEPES buffer supplemented with 10% glycerol. Protein purity was assessed by Coomassie brilliant blue staining. Purified proteins were concentrated using ultrafiltration units (Millipore, Waltham, MA, USA) and exchanged into PBS containing 10% glycerol for subsequent experiments.

**scRNA-seq Data Processing (human)**

A subset of the GSE173706 scRNA-seq dataset, comprising two peripheral normal skin samples (GSM5277178 and GSM5277181) and three psoriatic lesional skin samples (GSM5277189, GSM5277190, and GSM5277191), was processed using Seurat (v4.2.0). Cells with 200–7000 detected genes and < 10% mitochondrial reads were retained, merged across samples, normalized, and 2,000 highly variable genes were selected. Data were scaled with regression of cell-cycle effects and the number of detected genes, and batch effects were corrected using Harmony. PCA was performed followed by UMAP using the top 30 PCs, and clusters were identified by SNN-based Louvain clustering (resolution = 0.6). Cell types were annotated based on canonical marker genes (e.g., *MS4A1* for B cells, *CD3D/CD4/CD8B* for T cells, and *FOXP3* for regulatory T cells). Differentially expressed genes were identified using FindAllMarkers (|log_2_FC| > 0.25, adjusted *P* < 0.05), followed by GO/KEGG enrichment analysis using clusterProfiler. Furthermore, cell–cell communication analysis was performed using CellChat (v2.1.2) on the Normal and Psoriatic datasets following normalization. Overexpressed genes and ligand receptor interactions were identified using the built-in human ligand receptor database (*P* < 0.05), and communication probabilities were computed with computeCommunProb (nboot = 100, k = 0.5; *P* < 0.05). Results were summarized at the pathway level using computeCommunProbPathway, filtered to cell groups with ≥ 5 cells, and networks were aggregated and visualized using aggregateNet.

**scRNA-seq Data Processing (mouse)**

Public mouse skin single-cell RNA-seq data were obtained from GEO dataset GSE149121, including three control samples (GSM4490718, GSM4490719, and GSM4490720) and three IMQ-treated samples (GSM4490721, GSM4490722, and GSM4490723). Raw count matrices were analyzed using Seurat in R. Cells with fewer than 200 or more than 6000 detected genes, mitochondrial transcript proportions > 20%, or total UMI counts > 50,000 were excluded, and genes detected in fewer than three cells were removed. After normalization, variable-feature selection, scaling, and principal component analysis, clustering and UMAP visualization were performed using the first 20 principal components. Cell types were annotated based on canonical markers, including *Krt14/Krt5/Krt10/Krt1* for keratinocytes, *Dcn/Lum/Col1a1/Col3a1* for fibroblasts, *Adgre1/Cd68/C1qa/C1qb* for macrophages, *Cd3d/Cd3e* for T cells, *S100a8/S100a9/Ly6g* for neutrophils, *Pecam1/Kdr/Cdh5* for endothelial cells, and *Kit/Mcpt4/Cma1* for mast cells. Macrophage clusters were identified based on *Adgre1, Cd68, C1qa,* and *C1qb* expression, and the proportion of *Tmem173*-positive macrophages was calculated for each biological sample as the percentage of macrophages with detectable *Tmem173* expression. Sample-level values were compared between control and IMQ-treated groups using Welch’s two-tailed *t*-test.

**IL-23-induced psoriasis-like ear inflammation model**

Male and female C57BL/6J mice were used to establish an IL-23-induced psoriasis-like ear inflammation model. Mice were anesthetized with isoflurane and intradermally injected into the ear skin with recombinant mouse IL-23 (BioLegend, San Diego, CA, USA; Cat. No. 589005) at 500 ng in 10 μL PBS per ear every other day from Day 1 to Day 13. Control mice received an equal volume of PBS on the same schedule. PF treatment was initiated on Day 3 and administered intraperitoneally twice daily at 25 or 50 mg/kg per injection from Day 3 to Day 13, corresponding to total daily doses of 50 or 100 mg/kg, respectively. Methotrexate (MTX, 2 mg/kg, i.p., every other day) was used as a positive control. Ear thickness was measured at the indicated time points using a digital caliper. At the end of the experiment, mice were euthanized, and ear tissues were collected for histopathological examination and analysis of psoriasis-associated inflammatory markers. All animal experimental procedures were reviewed and approved by the Institutional Animal Care and Use Committee of Peking University Health Science Center (Animal Protocol No. DLASBF0549) and were conducted in accordance with institutional guidelines for animal welfare and research ethics.

**Generation and treatment of bone marrow-derived dendritic cells**

Bone marrow cells were isolated from the femurs and tibias of 4-week-old ICR mice and cultured in DMEM supplemented with 10% fetal bovine serum, 1% penicillin–streptomycin, recombinant mouse GM-CSF at 20 ng/mL (PeproTech, Rocky Hill, NJ, USA), and recombinant mouse IL-4 at 10 ng/mL (PeproTech, Rocky Hill, NJ, USA) for 7 days to generate BMDCs. Differentiated BMDCs were treated with PF at the indicated concentrations and LPS at 1 μg/mL for 24 h, after which *Il23a* mRNA expression and IL-23 secretion were assessed by RT–qPCR and ELISA, respectively.

**Zebrafish analysis**

Zebrafish embryos were maintained according to a standard protocol and raised to 3 days post-fertilization (dpf). At 3 dpf, transgenic Tg (lyz: DsRed2) zebrafish embryos with normal morphology were selected under a stereomicroscope. The embryos were randomly allocated to 6-well plates (10 embryos per well) and assigned to the following experimental groups: control, CuSO₄ (10 µM), PF at concentrations of 10, 25, or 50 μM, and ibuprofen as a positive control. PF was preincubated with embryos for 6 h, followed by exposure to CuSO₄ (10 µM) for 1 h to induce inflammatory injury. Neutrophil recruitment was assessed by capturing fluorescence images, and DsRed-positive neutrophils were quantified in the designated regions. All zebrafish experiments were conducted in accordance with relevant animal care and use guidelines and were approved by the Animal Ethical and Welfare Committee (AEWC) (Approval No. 202403).

**Enzyme-linked immunosorbent assay (ELISA)**

The serum concentrations of IL-23, IL-1β, IFN-γ and TGF-β1 were measured by ELISA Kits purchased from BioLegend (San Diego, CA, USA). Briefly, serum samples from IMQ-induced mice were collected by centrifugation and stored at −80 °C for subsequent analysis. Standards and diluted serum samples were added in duplicate, followed by sequential incubation with detection antibody and streptavidin–HRP, TMB substrate development, and reaction termination with stop solution. Absorbance was measured at 450 nm (570 nm reference), and concentrations were calculated from standard curves.

**Cell painting analysis**

RAW264.7 cells were plated in 96-well dishes and treated with LPS (1 μg/mL) and PF (100 μM) for 24 h. The cell painting dyes included: Hoechst 34580 (DNA/nucleus), concanavalin A, Alexa Fluor™ 488 Conjugate (ER), SYTO14 (RNA, nucleolus), Alexa Fluor™ 568 Phalloidin (F-actin), Wheat Germ Agglutinin, Alexa Fluor™ 555 Conjugate (Golgi) and MitoTracker™ Deep Red FM (mitochondria). Plates were imaged on a CV8000 high-content screening system (Yokogawa Electric Corporation, Musashino, Tokyo, Japan) with sequential imaging of six Cell Painting channels. Image analysis was performed using CellPathfinder (Yokogawa Electric Corporation, Musashino, Tokyo, Japan).

**RNA-seq and KEGG pathway enrichment analysis**

iBMDM cells were pretreated with or without PF (100 μM) for 18 h, followed by incubation with or without 2′,3′-cGAMP (cGAMP, 5 μM) for 6 h. Four experimental groups were included: vehicle control, PF alone, cGAMP alone, and cGAMP combined with PF. Total RNA was then extracted for transcriptomic analysis. RNA-seq library construction, sequencing, and initial data processing were performed by Novogene (Beijing, China). High-quality RNA samples were sequenced on an Illumina platform according to the manufacturer’s protocols. Differentially expressed genes were identified using standard statistical criteria and subjected to Kyoto Encyclopedia of Genes and Genomes (KEGG) pathway enrichment analysis to identify PF-associated signaling pathways.

**Agarose gel electrophoresis**

Agarose gels (2%) were prepared by dissolving 2 g agarose in 100 mL of TAE running buffer, heating until fully melted, cooling slightly, adding GelRed stain (1:10,000), and casting the gel in casting trays. PCR products generated by RT-PCR using KOD polymerase and the indicated mRNA primers were mixed with loading dye and loaded into each well. The samples were then separated by agarose gel electrophoresis at 100 V at room temperature until the bromophenol blue dye front reached the edge of the gel (approximately 60 min), and the bands were visualized under UV illumination. Images were acquired using a Tanon 1600 gel imaging system (Tanon Science & Technology, Shanghai, China).

**Microscale thermophoresis (MST) assay**

MST assays were performed using a Monolith NT.115 instrument (NanoTemper Technologies, Munich, Germany) to evaluate the binding of PF to wild-type (WT) and mutant STING proteins. HEK293T cells were transfected with plasmids encoding GFP-tagged WT STING or the indicated STING mutants using polyethylenimine (PEI) and incubated for 48 h. Cell lysates were incubated with increasing concentrations of PF (0.39–200 μM) for 30 min at room temperature and loaded into standard capillaries (NanoTemper Technologies, Munich, Germany). MST measurements were acquired using 50% LED power and 40% IR laser power, with a laser-on time of 30 s and a laser-off time of 5 s. Binding affinities (*K*_D_) were calculated using MO. Affinity Analysis software (NanoTemper Technologies, Munich, Germany).

**Circular dichroism (CD) analysis**

CD spectra were acquired using a JASCO J-810 spectropolarimeter (JASCO, Hachioji, Tokyo, Japan). Measurements were performed at 25 °C using 10 μM recombinant mouse STING protein (residues 149–378) in a 1-mm path-length quartz cuvette over a wavelength range of 197–260 nm. Spectra were acquired with a bandwidth of 1 nm, a data interval of 0.1 nm, a scanning speed of 50 nm/min, a digital integration time of 4 s, and three accumulations. CD spectra were collected in the absence or presence of increasing concentrations of PF (6.25–200 μM). To correct for PF-related background signals, CD spectra of PF alone at corresponding concentrations were subtracted as baselines. Data were analyzed using GraphPad Prism 8.0 (San Diego, CA, USA).

**Tryptophan fluorescence assay**

Intrinsic tryptophan fluorescence of STING was measured using a fluorescence spectrophotometer (PerkinElmer, Waltham, MA, USA). Fluorescence spectra were recorded with an excitation wavelength of 280 nm and emission collected from 300 to 650 nm. Recombinant mouse STING protein (residues 149–378; 5 μM) was added to a quartz-bottom 96-well plate, and fluorescence intensity was measured in the absence or presence of increasing concentrations of PF (3.18–25 μM). Normalized fluorescence data were analyzed using GraphPad Prism 8.0 (San Diego, CA, USA).

**Drug affinity responsive target stability (DARTS)**

RAW264.7 and THP-1 cells were lysed in NP-40 lysis buffer to prepare whole-cell lysates. The resulting lysates were incubated with PF (10, 50, 100 μM) for 1 h at 4 °C. Pronase was added at an optimized enzyme-to-protein ratio and incubated for 20 min at room temperature. Proteolysis was terminated by adding SDS–PAGE loading buffer, and protein stability was analyzed by Western blot.

**Identification of PF-binding peptides on STING**

Recombinant mouse STING (residues 149–378; 0.1 mg/mL) was incubated with PF (100 μM) or vehicle for 1 h at 4 °C, followed by digestion with pronase (10 μg/mL) for 20 min at room temperature. Proteolysis was terminated by the addition of SDS–PAGE loading buffer and heating at 98 °C for 10 min. Samples were separated by SDS–PAGE, and protein bands were excised for LC–MS/MS analysis.

**Cellular thermal shift assay (CETSA)**

RAW264.7 cells were lysed in PBS buffer and incubated with PF (100 μM) or vehicle for 1 h at 4 °C. Lysates were then aliquoted and heated at the indicated temperatures (37–82 °C) for 3 min, followed by cooling and centrifugation at 12,000 rpm for 15 min. The soluble fractions were analyzed by immunoblotting.

**Sequence comparison of human and mouse STING**

Canonical human STING (UniProt Q86WV6) and mouse STING (UniProt Q3TBT3) sequences were obtained from UniProt and aligned using EMBOSS Needle with the EBLOSUM62 matrix (gap-opening penalty, 10.0; gap-extension penalty, 0.5). Full-length STING and the cyclic dinucleotide-binding domains (human, amino acids 139–336; mouse, amino acids 138–335) were analyzed separately. Sequence identity and similarity are summarized in Supplementary Table 7.

**Molecular docking of PF with STING and protein–protein docking of STING with OGT**

Molecular docking of PF with human STING was performed using Schrödinger 2018. The crystal structure of human STING (PDB: 4KSY) was obtained from the Protein Data Bank and prepared using the Protein Preparation Wizard, followed by restrained minimization with the OPLS3 force field. PF was prepared using LigPrep. Induced-fit docking was performed using Glide XP precision. The docking score, key interacting residues, and corresponding interaction parameters are summarized in Supplementary Table 8.

Protein–protein docking between STING and OGT was performed using the HDOCK server. The AlphaFold-predicted structures of human STING (AF-Q86WV6-F1) and OGT (AF-O15294-F1) were used as the initial structures for docking. The top-ranked STING–OGT docking model was selected for subsequent structural analysis and molecular dynamics simulations. The HDOCK score and confidence score are summarized in Supplementary Table 9.

**Molecular dynamics (MD) simulations of the STING dimer in the presence or absence of PF**

Molecular dynamics simulations of the human STING dimer in the absence or presence of PF were performed using AMBER 22 with the STING dimer structure (PDB: 4EMT) as the initial protein model. Molecular mechanical parameters for PF were generated using Gaussian 16. Geometry optimization and electrostatic potential calculations were performed at the B3LYP/6-31G* level, followed by RESP charge fitting. The systems were solvated in an octahedral TIP3P water box with a 10 Å buffer and neutralized with Na^+^ counterions. Energy minimization was performed using 500 steps of steepest descent followed by 500 steps of conjugate-gradient minimization with positional restraints of 100 kcal mol^-1^ Å^-2^ on the solute atoms, followed by an additional 10,000 steps of energy minimization. The systems were then heated from 100 to 300 K over 200 ps, followed by 1 ns NVT and 500 ps NPT equilibration. Unrestrained production simulations were performed for 1 μs at 300 K and 1 atm using the PMEMD module of AMBER 22. The final frames of the MD trajectories were used for structural analysis.

**MD simulation of STING and Thr229-O-GlcNAcylated STING**

MD simulations of STING and Thr229-O-GlcNAcylated STING were performed using AMBER 22 with the AlphaFold-predicted human STING structure (AF-Q86WV6-F1) as the initial model. Molecular mechanical parameters for the Thr229 O-GlcNAc modification were generated using Gaussian 16. Geometry optimization and electrostatic potential calculations were performed at the B3LYP/6-31G* level, followed by RESP charge fitting. The systems were solvated in an octahedral TIP3P water box with a 10 Å buffer. Energy minimization was initially performed using 500 steps of steepest descent followed by 500 steps of conjugate-gradient minimization with positional restraints of 100 kcal mol⁻¹ Å⁻² on the solute atoms, followed by an additional 10,000 steps of energy minimization. The systems were then heated from 100 to 300 K over 200 ps, followed by 1 ns of NVT equilibration and 500 ps of NPT equilibration. Unrestrained production simulations were subsequently performed for 500 ns at 300 K and 1 atm using the PMEMD module of AMBER 22. The SHAKE algorithm was applied to bonds involving hydrogen atoms, and a 10 Å cutoff was used for Lennard–Jones interactions. The final frames of the MD trajectories were used for structural analysis.

**Figures**

**Figure S1.** **Single-cell analyses of macrophage involvement and cellular effects of PF in psoriasis** **(related to Figure 1).** **(A)** UMAP visualization of human skin single-cell RNA-seq data from normal and psoriatic lesional skin samples (GSE173706), colored by sample type. **(B)** UMAP visualization of scRNA-seq data from three control and three IMQ-treated mice, with major cell populations annotated. The relative proportions of individual cell types are shown based on the annotated clusters. Data were obtained from the publicly available GEO dataset GSE149121 (control: GSM4490718, GSM4490719, and GSM4490720; IMQ: GSM4490721, GSM4490722, and GSM4490723). **(C)** Circle plot of the interaction strength between macrophages and fibroblasts, keratinocytes, T cells, and natural killer T cells in normal and psoriatic skin. **(D)** CCK-8 assay of HaCaT cell viability following treatment with the indicated concentrations of PF (n = 6). **(E)** ELISA analysis of IL-23 secretion in BMDCs stimulated with LPS (1 μg/mL) and treated with the indicated concentrations of PF (n = 3). **(F)** RT-qPCR analysis of *Il23a* mRNA expression in BMDCs stimulated with LPS (1 μg/mL) and treated with the indicated concentrations of PF (n = 3). Data are presented as mean ± SEM. Statistical significance was determined by one-way ANOVA followed by Dunnett’s multiple comparisons test.

**Figure S2.** **PF effectively suppresses macrophage-mediated inflammation in psoriasis** **(related to Figure 1).** **(A)** Representative fluorescence images and quantification of neutrophils in a CuSO_4_-induced zebrafish inflammation model following PF or ibuprofen treatment (n = 6). Scale bars, 200 μm. **(B)** Representative flow cytometry plots of CD11b^+^F4/80^+^ macrophages among splenic CD45^+^ cells in each group (n = 5). **(C)** Schematic illustration of the IL-23-induced psoriasis-like ear inflammation model and treatment regimen, with representative ear images at the experimental endpoint. **(D)** Ear thickness of mice in the indicated groups on day 13 (n = 6). **(E)** Representative H&E-stained ear skin sections with quantification of epidermal thickness (n = 3). Scale bars, 25 μm. Data are presented as mean ± SEM. Statistical significance was determined by one-way ANOVA followed by Dunnett’s multiple comparisons test.

**Figure S3. Validation of STING as a direct target of PF (related to Figure 2). (A)** Workflow for synthesizing PF beads. **(B-D)** Immunoblot analysis of STING, TLR2, and TLR7 in RAW264.7 cells transfected with the indicated siRNAs, with non-targeting siRNA as the negative control. **(E)** RT-qPCR analysis of *Il6, Ptgs2, Ccl2,* and *Il23a* expression in siTLR2-transfected RAW264.7 cells treated with LPS (1 μg/mL) with or without PF (100 μM) (n = 3). **(F)** RT-qPCR analysis of *Il6, Ptgs2, Ccl2,* and *Il23a* expression in siTLR7-transfected RAW264.7 cells treated with LPS (1 μg/mL) with or without PF (100 μM) (n = 3). **(G)** Immunoblot analysis of DHX9 expression in RAW264.7 cells transfected with DHX9 siRNAs. **(H)** RT-qPCR analysis of *Il6* and *Il23a* expression in siDHX9-transfected RAW264.7 cells treated with LPS (1 μg/mL) with or without PF (100 μM) (n = 3). **(I)** DARTS analysis of endogenous STING stability in THP-1 cell lysates treated with PF, with quantification (n = 3). **(J)** Violin plot of *Tmem173* (STING) expression in macrophages from control and IMQ-treated mouse skin single-cell RNA-seq data (GSE149121). The *P* value was calculated by comparing sample-level proportions of *Tmem173*-positive macrophages using Welch’s two-tailed t-test (n = 3). Data are presented as mean ± SEM. Statistical significance was determined by one-way ANOVA followed by Dunnett’s multiple comparisons test. For comparisons involving NC and siRNA groups, two-way ANOVA followed by Dunnett’s multiple comparisons test was used.

**Figure S4. Mutational analysis of key residues involved in PF binding to hSTING (related to Figure 3). (A)** Representative Sanger sequencing chromatograms of GFP-hSTING-WT and GFP-hSTING-4A constructs. The E174A, T181A, Q227A, and T229A substitutions are highlighted by red dashed boxes. **(B)** MST analysis of PF binding to GFP-tagged hSTING-WT and hSTING-4A (n = 3).

**Figure S5. PF stabilizes STING in an inactive open conformation (related to Figure 3). (A)** Molecular docking analysis of PF binding to mouse STING (PDB: 4KC0). **(B)** Molecular dynamics simulation analysis of mouse STING (PDB: 4KC0) conformational changes in the presence or absence of PF. The major allosteric regions are highlighted by dotted rectangles, with representative local conformational changes shown in the inset panels. The right panel shows the structure after 180° rotation.

**Figure S6. PF suppresses cGAMP-induced STING trafficking to the Golgi (related to Figure 4). (A)** Immunoprecipitation analysis of STING *O*-GlcNAcylation in iBMDMs transfected with HA-STING and treated with Thiamet-G (10 μM) in the presence or absence of PF (100 μM). **(B)** Immunoblot analysis of global *O*-GlcNAcylation levels in iBMDMs stimulated with cGAMP and treated with the indicated concentrations of PF. Relative protein levels were quantified and normalized to GAPDH (n = 3). **(C)** DARTS analysis of endogenous OGT in THP-1 cell lysates treated with PF, with quantification (n = 3). **(D)** Confocal microscopy analysis of GFP-STING and mCherry-Golgi colocalization in HEK293T cells (n = 3). Scale bars, 10 μm. **(E)** Cell Painting analysis of RAW264.7 cells treated with LPS (1 μg/mL) with or without PF (100 μM), with quantification of average organelle fluorescence intensity (n = 3). Scale bars, 6 μm. Data are presented as mean ± SEM. Statistical significance was determined by one-way ANOVA followed by Dunnett’s multiple comparisons test.

**Figure S7. STING *O*-GlcNAcylation at Thr229 modulates distal Ser366 phosphorylation (related to Figure 5). (A)** Immunoblot analysis of OGT protein levels and agarose gel electrophoresis analysis of Ogt RT-PCR products in iBMDMs following CRISPR–Cas9-mediated gene editing. Relative protein levels were quantified, normalized to GAPDH, and expressed relative to the negative control (NC) group (n = 3). **(B)** Immunoblot analysis of STING protein levels in HEK293T cells after CRISPR–Cas9-mediated gene editing. Relative protein levels were quantified, normalized to GAPDH, and expressed relative to the NC group (n = 3). **(C)** Immunoblot analysis of STING protein levels in iBMDMs after CRISPR–Cas9-mediated gene editing. Relative protein levels were quantified, normalized to GAPDH, and expressed relative to the NC group (n = 3). **(D)** Sanger sequencing analysis of STING-WT and STING-T229A constructs. **(E)** Immunofluorescence analysis of p-STING and global *O*-GlcNAcylation in iBMDMs under the indicated treatments (n = 3). Scale bars, 10 μm. Data are presented as mean ± SEM. Statistical significance was determined by one-way ANOVA followed by Dunnett’s multiple comparisons test.

**Figure S8. PF attenuates STING-dependent TBK1-NF-κB signaling axis activation (related to Figure 6). (A)** Immunoblot analysis of STING and TBK1 phosphorylation in RAW264.7 cells treated with LPS (1 μg/mL) and the indicated concentrations of PF. Band intensities were quantified by ImageJ (n = 3). **(B)** GSEA of RNA-seq data from iBMDMs treated with cGAMP alone or in combination with PF. Data are presented as mean ± SEM. Statistical significance was determined by one-way ANOVA followed by Dunnett’s multiple comparisons test.

**Figure S9. PF suppresses downstream NF-κB activation (related to Figure 6). (A)** Principal component analysis (PCA) of RNA-seq samples (n = 3). **(B)** Volcano plots of differential gene expression (n = 3). **(C)** Immunoblot analysis of IKKα/β, IκBα, and NF-κB p65 phosphorylation in LPS-stimulated RAW264.7 cells treated with PF. Band intensities were quantified by ImageJ (n = 3). **(D)** Representative immunofluorescence images of NF-κB p65 nuclear localization in LPS-stimulated RAW264.7 cells treated with PF (n = 3). Scale bars, 20 μm. Data are presented as mean ± SEM. Statistical significance was determined by one-way ANOVA followed by Dunnett’s multiple comparisons test.

**Figure S10.** **Myeloid-specific STING deficiency inhibits psoriasis progression in mice (related to Figure 7). (A)** Genotyping analysis of toe DNAs from Lyz2-Cre^+/-^Sting^+/+^ (WT), Lyz2-Cre^+/-^Sting^fl/+^ (Het), Lyz2-Cre^+/-^Sting^fl/fl^ (cKO). PCR screening of the *Sting* gene revealed a band of ~450 bp for the floxed allele and 298 bp for the wild-type allele. **(B)** PCR analysis of *Lyz2*-Cre genotyping, with a ~700 bp band for the mutant allele and a 350 bp band for the wild-type allele. Lyz2-Cre^+/−^ mice exhibited both bands. **(C)** Immunoblot analysis of STING protein levels in BMDMs from WT and STING cKO mice. Band intensities were quantified by ImageJ (n = 3). **(D)** RT-qPCR analysis of *Sting* expression in BMDMs from WT and STING cKO mice (n = 5). **(E)** Representative multiplex immunofluorescence images of STING, F4/80, Ly6G, and Ly6C in lesional skin from WT and STING cKO mice. Nuclei were counterstained with DAPI. Scale bars, 25 μm. **(F)** Body weight of 10-week-old male WT and cKO mice (n = 6). **(G)** IHC analysis of *O*-GlcNAc in lesional skin from WT and STING cKO mice after IMQ induction and PF treatment (n = 3). Scale bars, 100 μm. Data are presented as mean ± SEM. Statistical significance was determined by one-way ANOVA followed by Dunnett’s multiple comparisons test.

**Figure S11.** **OGT expression and inflammatory gene signatures in psoriasis patients (related to Figure 8). (A)** Violin plot of OGT expression across different cell populations in normal and psoriatic human skin single-cell RNA-seq data (GSE173706). **(B)** RT-qPCR analysis of *IFNB1* expression in whole-blood samples from healthy individuals and patients with psoriasis (n = 12). **(C)** RT-qPCR analysis of the indicated genes in whole-blood samples from healthy individuals and patients with psoriasis (n = 12). Data are presented as mean ± SEM. Statistical significance was determined using two-tailed unpaired Student’s *t*-tests.

**Supplementary Table 1. Information on the major chemicals and reagents used in this study**

| **Reagent** | **Supplier** | **Catalog No.** | **Purity** |
| --- | --- | --- | --- |
| Paeoniflorin (PF) | TargetMol | T2230 | 97.43% |
| 2′,3′-cGAMP (cGAMP) | MedChemExpress | HY-100564 | 99.95% |
| Imiquimod (IMQ) | Sichuan Mingxin Pharmaceutical | H20030128 | 5% (w/w) |
| OSMI-4 | MedChemExpress | HY-114361 | 99.69% |
| Thiamet-G | MedChemExpress | HY-12588 | 99.99% |
| Methotrexate (MTX) | BLDpharm | BD430228 | 99.99% |

**Supplementary Table 2.** **Mouse genotyping primers used in this study.**

| **Name** | **Sequence (5’-3’)** |
| --- | --- |
| Sting1-flox-F | TTTTCATCTGCCTTCCAGGT |
| Sting1-flox-R | GCGCACACACACTAAAAACTG |
| Lyz2-Cre-oIMR3066 (Mut) | CCCAGAAATGCCAGATTACG |
| Lyz2-Cre-oIMR3067 (common) | CTTGGGCTGCCAGAATTTCTC |
| Lyz2-Cre-oIMR3068 (WT) | TTACAGTCGGCCAGGCTGAC |

**Supplementary Table 3. Primer sequences used for RT-qPCR in this study.**

| **Name** | **Sequence (5’-3’)** |
| --- | --- |
| *Human STING* | F: CCTGAGTCTCAGAACAACTGCC |
|  | R: GGTCTTCAAGCTGCCCACAGTA |
| *Human OGT* | F: CTTTAGCACTCTGGCAATTAAACAG |
|  | R: TCAAATAACATGCCTTGGCTTC |
| *Human IFNB1* | F: GTCTCCTCCAAATTGCTCTC |
|  | R: ACAGGAGCTTCTGACACTGA |
| *Human CXCL1* | F: CAATCCTGCATCCCCCATAGT |
|  | R: GGATTTGTCACTGTTCAGCATCTT |
| *Human CXCL2* | F: AGATCAATGTGACGGCAGGG |
|  | R: TCTCTGCTCTAACACAGAGGGA |
| *Human CXCL8* | F: AACTGAGAGTGATTGAGAGTGG |
|  | R: ATGAATTCTCAGCCCTCTTCAA |
| *Human IL17A* | F: TCCCACGAAATCCAGGATGC |
|  | R: GGATGTTCAGGTTGACCATCAC |
| *Human IL23A* | F: CTCAGGGACAACAGTCAGTTC |
|  | R: ACAGGGCTATCAGGGAGCA |
| *Human TNF* | F: GAGGCCAAGCCCTGGTATG |
|  | R: CGGGCCGATTGATCTCAGC |
| *Human GAPDH* | F: GGAGCGAGATCCCTCCAAAAT |
|  | R: GGCTGTTGTCATACTTCTCATGG |
| *Mouse Sting* | F: GGTCACCGCTCCAAATATGTAG |
|  | R: CAGTAGTCCAAGTTCGTGCGA |
| *Mouse Cxcl1* | F: CTGGGATTCACCTCAAGAACATC |
|  | R: CAGGGTCAAGGCAAGCCTC |
| *Mouse Cxcl10* | F: CCAAGTGCTGCCGTCATTTTC |
|  | R: GGCTCGCAGGGATGATTTCAA |
| *Mouse Il6* | F: CACAGAAGGAGTGGCTAA |
|  | R: CCATAACGCACTAGGTTT |
| *Mouse Tnf* | F: AAGCAAGCAGCCAACCAG |
|  | R: CCACAAGCAGGAATGAGAAGA |
| *Mouse Ptgs2* | F: GCGACATACTCAAGCAGGAGCA |
|  | R: AGTGGTAACCGCTCAGGTGTTG |
| *Mouse Ccl2* | F: TTAAAAACCTGGATCGGAACCAA |
|  | R: GCATTAGCTTCAGATTTACGGGT |
| *Mouse Il23a* | F: TGGAGCAACTTCACACCTCC |
|  | R: GGCAGCTATGGCCAAAAAGG |
| *Mouse Il17a* | F: TCTCCACCGCAATGAAGACC  R: TTTCCCTCCGCATTGACACA |
| *Mouse Gapdh* | F: GGTGAAGGTCGGTGTGAACG |
|  | R: CTCGCTCCTGGAAGATGGTG |

**Supplementary Table 4. sgRNA target sequences used in this study.**

| **Name** | **Sequence (5’-3’)** |
| --- | --- |
| Human STING-sg-1 | GCTGGGACTGCTGTTAAACG |
| Human STING-sg-2 | CATATTACATCGGATATCTG |
| Human STING-sg-3 | TACTCCCTCCCAAATGCGGT |
| Mouse Sting-sg-1 | ATATTTGGAGCGGTGACCTC |
| Mouse Sting-sg-2 | GCTGGCCACCAGAAAGATGA |
| Mouse Sting-sg-3 | TGTAGCCCTCATCTTTCTGG |
| Mouse Ogt-sg-1 | GGCAACGTGGCCGACAGTAC |
| Mouse Ogt-sg-2 | CCGTGCACCACCGCATGATT |

**Supplementary Table 5. siRNA sequences used in this study.**

| **Target** | **Sequence (5’-3’)** |
| --- | --- |
| siSTING | Sense: GGAGCCGAAGACUGUACAUTT |
|  | Antisense: AUGUACAGUCUUCGGCUCCTT |
| siTLR7 | Sense: GGAGCACACAAAGGUCAAATT |
|  | Antisense: UUUGACCUUUGUGUGCUCCTT |
| siTLR2 | Sense: GGAACAGAGUGGCAACAGUTT |
|  | Antisense: ACUGUUGCCACUCUGUUCCTT |
| siDHX9 | Sense: GCUGCCAGAGACUUUGUUATT |
|  | Antisense: UAACAAAGUCUCUGGCAGCTT |
| siNC | Sense: UUCUCCGAACGUGUCACGUTT |
|  | Antisense: ACGUGACACGUUCGGAGAATT |

**Supplementary Table 6. Antibodies used in this study.**

| **Antibody** | **Supplier** | **Catalog no.** | **Application** |
| --- | --- | --- | --- |
| Anti-STING antibody | Cell Signaling Technology | 13647 | WB (1:1000) |
| Anti-phospho-STING (Ser366) antibody | Abcam | 338079 | WB (1:1000) |
| Anti-phospho-STING (Ser366/Ser365) antibody | ABMART | P99064 | IHC (1:200) |
| Anti-TBK1 antibody | Cell Signaling Technology | 3013 | WB (1:1000) |
| Anti-phospho-TBK1 (Ser172) antibody | Cell Signaling Technology | 5483 | WB (1:1000) |
| Anti-O-GlcNAc antibody | Proteintech | 65292-1-Ig | WB (1:1000) |
| Anti-OGT antibody | Abcam | ab177941 | WB (1:1000) |
| Anti-TLR2 antibody | Abcam | ab209217 | WB (1:1000) |
| Anti-TLR7 antibody | Abcam | ab113524 | WB (1:1000) |
| Anti-DHX9 antibody | Aladdin | Ab125456 | WB (1:1000) |
| Anti-HA tag antibody | Proteintech | 51064-2-AP | WB (1:5000) |
| Anti-FLAG tag antibody | Proteintech | 66008-4-Ig | WB (1:5000) |
| Anti-MYC tag antibody | Proteintech | 60003-2-Ig | WB (1:5000) |
| Anti-IKKβ antibody | Cell Signaling Technology | 8943 | WB (1:1000) |
| Anti-phospho-IKKα/β (Ser176/180) antibody | Cell Signaling Technology | 2697 | WB (1:1000) |
| Anti-IκBα antibody | Cell Signaling Technology | 4814 | WB (1:1000) |
| Anti-phospho-IκBα (Ser32) antibody | Cell Signaling Technology | 2859 | WB (1:1000) |
| Anti-NF-κB p65 antibody | Cell Signaling Technology | 8242 | WB (1:1000) |
| Anti-phospho-NF-κB p65 (Ser536) antibody | Cell Signaling Technology | 3033 | WB (1:1000) |
| Anti-GAPDH antibody | Proteintech | 60004-1-Ig | WB (1:5000) |
| PE anti-mouse CD45 antibody | BD Biosciences | 553081 | FC (1:100) |
| APC anti-mouse F4/80 antibody | BioLegend | 123116 | FC (1:100) |
| FITC anti-mouse/human CD11b antibody | BioLegend | 101205 | FC (1:100) |
| TruStain FcX™ PLUS anti-mouse CD16/32 antibody | BioLegend | 156604 | FC (1:100) |

**Supplementary Table 7. Sequence comparison between human and mouse STING**

| **Feature** | **Human STING** | **Mouse STING** | **Identity** | **Similarity** |
| --- | --- | --- | --- | --- |
| Full-length | aa 1–379 | aa 1–378 | 68.9% | 81.0% |
| CBD | aa 139–336 | aa 138–335 | 79.3% | 87.9% |

**Supplementary Table 8. Information on the molecular docking score and interaction parameters of PF with STING**

| **Complex** | **Structure** | **Docking score** | **interacting residues** | **Interaction distances (Å)** |
| --- | --- | --- | --- | --- |
| PF–STING | PDB: 4KSY | −6.434 | E174, T181, Q227, T229 | 2.78, 2.89, 2.90, 2.99, 3.35 |

**Supplementary Table 9. Information on the protein–protein docking parameters between STING and OGT**

| **Complex** | **Structures** | **Docking method** | **Selected model** | **HDOCK score** | **Confidence score** |
| --- | --- | --- | --- | --- | --- |
| STING–OGT | STING: AF-Q86WV6-F1; OGT: AF-O15294-F1 | HDOCK | Rank 1 | −327.04 | 0.9718 |
